# An NKX2-3/5/6 homolog is required downstream of BMP signaling to pattern the sensory-adhesive organ of a tunicate larva

**DOI:** 10.1371/journal.pgen.1012334

**Published:** 2026-10-12

**Authors:** Christopher J. Johnson, Joshua Kavaler, Timethea Slade, Kayla Wilson, Christina D. Cota, Alberto Stolfi

**Affiliations:** 1 School of Biological Sciences, Georgia Institute of Technology, Atlanta, Georgia, United States of America; 2 Biology Department, Colby College, Waterville, Maine, United States of America; Columbia University, UNITED STATES OF AMERICA

## Abstract

Tunicates are the sister group to vertebrates within the chordate phylum, yet unlike other chordate groups, they evolved a biphasic lifecycle alternating between motile larvae and sessile adults. The papillae of most tunicate larvae are the key sensory-adhesive organ regulating their settlement and metamorphosis. The papillae are nearly always arranged as a group of three morphologically identical organs that arise from an anterior neural plate border region nested between ventral epidermis and more dorsal/posterior neural tube progenitors. Due to their embryonic origin and molecular signatures, this anterior border has been evolutionarily linked to vertebrate placode regions. It was previously shown that the specification, patterning, and morphogenesis of the embryonic papilla region all depend on BMP signaling, though downstream mechanisms remain poorly understood. Here we show that the NKX2-3/5/6 ortholog NK4 is a key transcription factor that acts downstream of BMP signaling to pattern the papillae in the *Ciona* embryo. We present evidence that *NK4* is activated by BMP signaling and encodes a transcriptional repressor that is required to restrict the expression of the papilla regulatory gene *Foxg* to three cell clusters that give rise to the three papillae. Loss of NK4 function results in the formation of a single large papilla. In contrast, overexpression of NK4 represses *Foxg,* eliminating the papillae. We also show that the expression of *NK4* is restricted dorsally by the BMP antagonist Chordin, while the ventrally-expressed transcription factor Msx can alleviate the repressive effect of NK4, potentially allowing for the characteristic tripartite patterning of the papillae.

## Author summary

Many marine animals, like most of our close non-vertebrate chordate relatives the tunicates, begin life as swimming larvae that must find the right surface on which to attach before undergoing metamorphosis into the sedentary adult phase. This decision relies on sensory-adhesive organs called the papillae, which frequently appear in a set of three matching parts arranged in a triangle. How an embryo builds exactly three identical papilla organs, rather than one continuous, disorganized patch of tissue, has remained unclear. Using the model tunicate *Ciona*, we show that a protein called NK4, homologous to human factors NKX2-3, NKX2-5, and NKX2-6, plays a central role in this process. NK4 is switched on by a signaling protein called BMP, which spreads across the region that will form the papillae. Once active, NK4 refines the activation pattern of another key factor, Foxg, so that only three small clusters of cells that go on to become the three papillae. When we removed NK4 using CRISPR gene editing, the three papillae fused into a single large one instead. Our results help explain how a uniform field of cells can reliably split into three separate organs.

## Introduction

Tunicates have emerged as model organisms for the study of chordate evolution and development [1]. Despite sharing key anatomical, cellular, and molecular traits with other chordate subphyla, a majority of tunicate species alternate between motile, pelagic larval and sessile, benthic adult lifecycle phases [2,3]. The sensory-adhesive papilla organ of the larva plays an indispensable role in driving substrate attachment and metamorphosis in most species [4]. In solitary species studied in the lab, it has been shown that continuous attachment to a solid substrate is necessary and sufficient to trigger metamorphosis [5]. This metamorphosis has been shown to be driven by the mechanosensory abilities of the papillae, and direct mechanical or optogenetic stimulation of the papilla neurons (PNs) can substitute for attachment in initiating metamorphosis [6,7]. It is thought that the PNs sense mechanical forces generated and/or augmented by substrate attachment [8], which in turn depends on adhesive material-secreting collocytes that are present alongside the PNs in the papilla organ [9,10]. Consistent with this model, metamorphosis is blocked by genetically eliminating PNs or perturbing their function [11]. In most species, the papilla organ is invariably organized in a tripartite manner, consisting of three constituent papillae arranged as vertices of a triangle: two dorsal, lateral papillae and one ventral, medial papilla [12–14]. Each papilla appears to consist of the same number and arrangement of cell types, of which at least five are known based on cytological and molecular characterization [10,15]. This tripartite arrangement is highly conserved, even in the much larger larvae of certain compound tunicate species [16], suggesting some adaptive advantage for the larva.

The papillae arise from the anteriormost cells of the neural plate, forming an anterior neural border (ANB) that might be evolutionarily connected to the anterior cranial placodes of vertebrates [17–26]. In multiple species studied, the specification and patterning of the papilla region is regulated by BMP signaling [20,21]. First, BMP signaling is required for proper papilla specification, though in more anterior/ventral epidermis it may suppress papilla fate [20,21]. Later, BMP signaling patterns the papilla territory into its familiar tripartite arrangement [20,21]. It has also been shown that the tripartite organization of the papillae depends on the precise specification of three discrete, Foxg + /Islet+ cell clusters at the mid/late tailbud stages [15,21,27,28]. However, our understanding of how BMP or other candidate pathways accomplish this remains incomplete (Fig 1).

**Fig 1:**
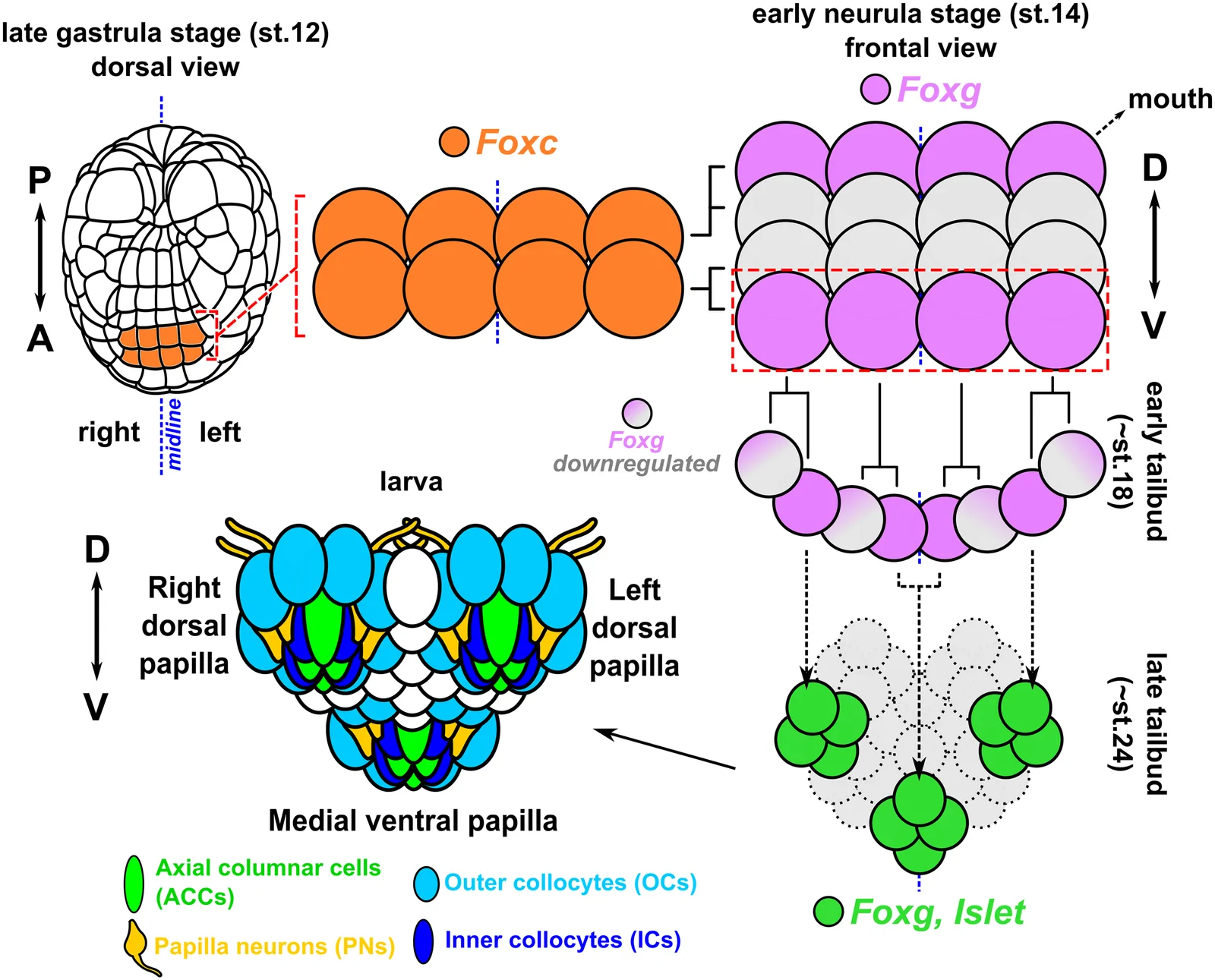
Summary diagram of papilla development in *Ciona.* A stylized diagram summarizing what is currently know about the cell lineages and major cell fate specification events in the developing papilla territory of *Ciona* embryos. Briefly, the papillae are derived from anterior-most *Foxc*-expressing cells of the anterior neural plate border at gastrula stage (st.12). The anterior-most row of daughter cells (red dashed rectangle) go on to express *Foxg* at the early neurula stage (st.14). During tailbud stages, this row of cells begins to bend ventrally, forming a “U”-shaped swatch of cells. *Foxg* expression is maintained in three discrete clusters of cells and downregulated in the remaining cells of this row at st.18. Eventually, sustained Foxg activates *Islet* in the three cell clusters and their descendants, giving rise to the three protruding sensory-adhesive papillae of the hatched larva (st.24): one medial, one ventral papilla, and two lateral, dorsal papillae. Each papilla is composed of invariant numbers of different cell types (4 axial columnar cells, 4 inner collocytes, 4 papilla neurons, and 8 outer collocytes, for a total of 20 cells). See text for references. A: anterior, P: posterior, D: dorsal, V: ventral.

Here we show that the BMP-responsive regulatory gene *Nkx2–3/5/6,* the ortholog of *NKX2–3, NKX2–5,* and *NKX2–6* in humans, and of *tinman* in *Drosophila,* is a key intermediate regulator that mediates the BMP-dependent tripartite patterning of the *Ciona* papillae. We show that BMP signaling in the papilla territory promotes expression of *Nkx2–3/5/6* (which we call *NK4* herein, based on the original name of the *Drosophila* tinman protein, NK-4) [29]. We further show that NK4 acts as a transcriptional repressor that is both necessary and sufficient to downregulate *Foxg* expression in specific sub-regions of this territory, thereby allowing for the formation of discrete *Foxg+* cell clusters that give rise to the three papillae. Disrupting *NK4* largely phenocopies BMP inhibition, resulting in a single large papilla. We discuss our findings in the context of a larger model that we propose as underlying the development of the conserved tripartite organization of the papilla organ, and its implications for larval settlement and metamorphosis.

## Results

### Knocking out NK4 in the papillae using CRISPR/Cas9 phenocopies BMP inhibition

We initially identified NK4 as a potential regulator of cell fate in the papillae. *NK4* is expressed throughout the papilla territory (Figs 2 and S1 in S1 File and S1 Video) [32], where it was hypothesized to be important for the specification of the Axial Columnar Cells (ACCs), a papilla cell type with sensory capabilities but of unknown function [32,33]. We thus originally sought to knock out *NK4* specifically in the papilla territory, to assay its role in ACC specification. To do so, we selected a previously designed and validated, high-efficiency single-chain guide RNA (sgRNA) targeting the second exon of *NK4* (Fig 2B), predicted to cut within the sequence encoding part the DNA-binding homeodomain [34]. To limit CRISPR/Cas9 activity to the early papilla cell lineages, we used the *Foxc* promoter to drive expression of Cas9 (*Foxc>Cas9*) [15]. When we co-electroporated the sgRNA expression plasmid (*U6 > NK4.3*) with *Foxc>Cas9,* we unexpectedly observed a “*Cyrano*” phenotype [21] in which a single protruding papilla forms in place of the familiar three papillae arranged as a triangle (Fig 2C–2E). This phenotype was originally observed in larvae subjected to BMP signaling knockdown [20,21], but to our knowledge this is the first report of a CRISPR condition with the same or similar phenotype. The ACC reporter gene *CryBG > GFP* was still expressed in *NK4* CRISPR larvae showing a clear single-papilla phenotype (Fig 2C and 2F), suggesting that NK4 is specifically involved in papilla organ patterning but not necessarily cell type specification or differentiation. To test this, we replicated the CRISPR-mediated disruption of *NK4* while assaying the expression of reporters that label the other cell types of the papillae (Fig 3A and 3B). Indeed, all cell type reporters were still labeling cells contributing to the resulting single large papilla. The same phenotype of a single large papilla was obtained using another sgRNA targeting a different part of the *NK4* gene, further supporting our observations (S2 Fig in S1 File). Despite only having one large papilla, *NK4* CRISPR larvae were still fully capable of undergoing metamorphosis (Fig 3C and 3D), likely due to the fact that all the cell types, especially the PNs, were still present. Of note, PNs in *NK4 CRISPR* larvae still had their apical cilia, which are hypothesized to be important for mechanosensory ability (S2–S4 Videos) These results suggest that the tripartite organization of the papillae might not be required for metamorphosis under laboratory conditions, but could be more important for robust adhesion or to fine-tune substrate selection in the wild.

**Fig 2:**
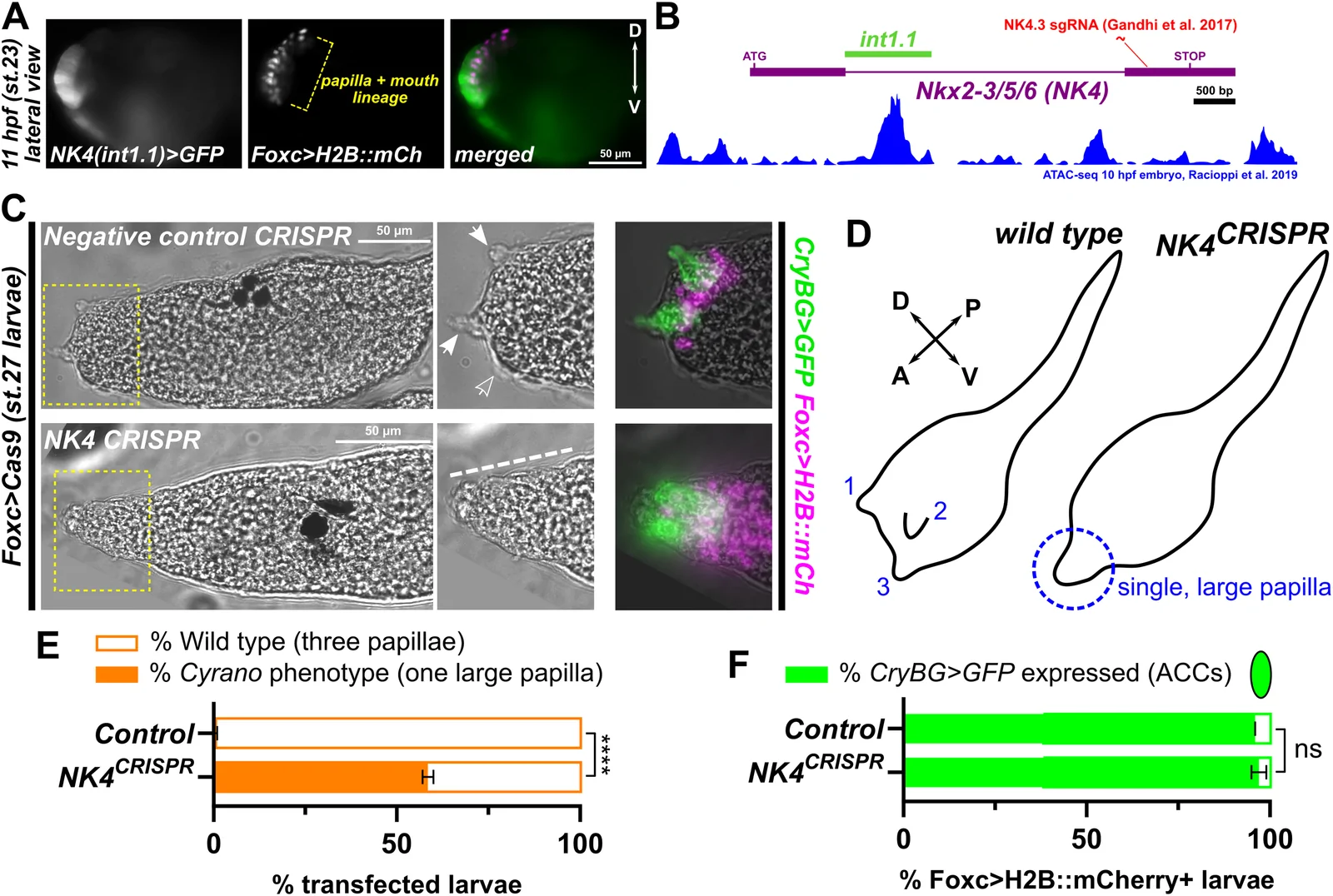
*NK4,* an *NKX2-3/5/6* ortholog, is required for the tripartite patterning of the papillae. **A)** A GFP reporter plasmid constructed using a portion of the sole intron of the *NK4* gene of *Ciona robusta* (“int1.1”)*,* fused to the basal promoter of the *FOG* gene [30]. See S1 Text for details), is expressed throughout the Foxc+ papilla territory in stage 23 embryos, recapitulating the previously reported mRNA *in situ* hybridization pattern. Int1.1 reporter activity can also be seen in ventral epidermis and trunk endoderm. hpf = hours post-fertilization. **B)** Diagram depicting the *NK4* locus and location of both the int1.1 *cis*-regulatory element and the target of the published *NK4.3* sgRNA. Underneath is the ATAC-seq profile of 10 hpf embryos, from Racioppi et al. 2019, showing a peak of DNA accessibility centered on the int1.1 element. **C)** Papilla lineage-specific CRISPR/Cas9-mediated mutagenesis of *NK4* showing the single, enlarged papilla protrusion (indicated by dashed line) similar to the *Cyrano* phenotype described in [21] and replicated in [20]. Compare to the negative control CRISPR using the non-targeting *“Control”* sgRNA instead, with two electroporated papillae (solid arrowheads) and one unelectroporated papilla (hollow arrowhead). Right panels: the *CryBG > GFP* reporter (green) reveals the presence of ACCs in the single papilla of the *NK4* CRISPR larvae. Left panels show brightfield images (zoomed in panel indicated in yellow dashed outline), while right panels show brightfield overlaid on GFP/mCherry fluorescence. **D)** Schematic diagram of wild type (negative control) larvae with three papillae, compared to *NK4* CRISPR larvae with a single large papilla A: anterior, P: posterior, D: dorsal, V: ventral. **E)** Scoring the percentage of transfected larvae showing the single large papilla phenotype (“*Cyrano*”) in *NK4* CRISPR vs. negative control larvae electroporated with only the reporter plasmids. Transfection was monitored using a combination of *Islet>GFP* or *CryBG > GFP* and *Foxc>H2B::mCherry* reporter plasmid expression in the papillae * p < 0.0001 for both duplicates per Fisher’s exact test. **F)** Scoring the proportion of transfected (*Foxc>H2B::mCherry+*) larvae showing *CryBG > GFP* expression as shown in panel C. No difference was observed in the proportion of larvae expressing *CryBG > GFP* between *NK4* CRISPR and control larvae. Experiment performed in duplicate ns = not statistically significant per Fisher’s exact test. All GFP sequences are tagged with Unc-76, which promotes cytoplasmic localization over nuclear localization [31]. Scale bars = 50 m. mCh: mCherry; D: dorsal; V: ventral. All experiments were performed in duplicate and averaged, with n = 100 per condition/duplicate, with error bars indicating upper and lower limit. See S1 Data for all scoring data and statistical tests.

**Fig 3:**
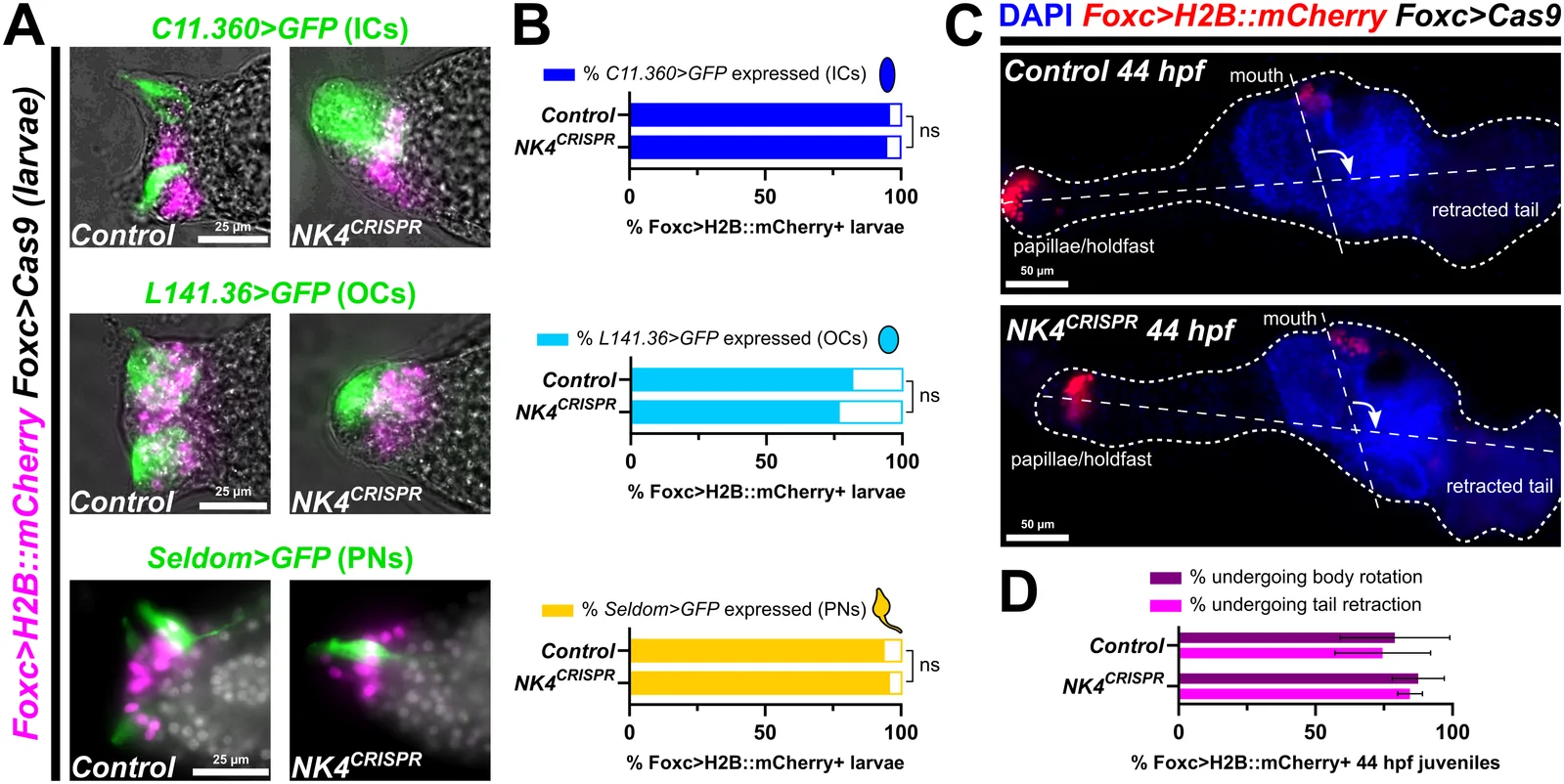
*NK4* CRISPR does not significantly impact specification of other papilla cell types or metamorphosis, despite resulting in a single large papilla. **A)** Papilla lineage-specific *CRISPR* mutagenesis of *NK4* does not result in loss of other papilla cell type-specific reporter gene expression in spite of the loss of the typical tripartite organization of the papillae. Experiment was performed in duplicate. ICs: inner collocytes; OCs: outer collocytes; PNs: papilla neurons. Counterstain in *Seldom>GFP* images is DAPI (white overlay). Scale bars = 25 µm. IC and PN images acquired at 17 hours post-fertilization (hpf; St. 27), while OC images were acquired at 21 hpf (St. 29) on account of the late onset of *L141.36* expression as previously reported [15]. **B)** Scoring the percentage of transfected larvae showing expression of each cell type-specific reporter (single replicate each), comparing *NK4* CRISPR vs. negative control larvae as shown in panel A. n = 100 for all conditions. ns = not statistically significant per Fisher’s exact test. **C)** *NK4* CRISPR in the papilla lineage also does not significantly prevent metamorphosis, as assayed in 44 hpf juveniles (St. 35) undergoing tail retraction and body axis rotation (white arrow). Scale bars = 50 µm. **D)** Scoring the percentage of transfected juveniles undergoing either metamorphic process as depicted in panel C. Experiment was performed in duplicate with n = 75-100 per condition/duplicate and averaged, with errors bars indicating upper and lower limits. Duplicate 1 did not yield statistically significant difference between control and *NK4* CRISPR, while Duplicate 2 showed a statistically significant increase in metamorphosis in the *NK4* CRISPR condition (all Fisher’s exact tests). All GFP sequences tagged with Unc-76. See S1 Data for all scoring data and statistical tests.

### BMP signaling activates *NK4* expression in the papilla territory

In *Ciona, NK4* has been shown to be activated by BMP signaling [21,35–37]. In the migrating cardiopharyngeal progenitors, BMP signaling activates *NK4,* which in turn modulates cell migration and fate decisions in this lineage [35]. In other studies, a ventral-to-dorsal gradient of BMP activity has been meticulously documented [21,37], largely overlapping with *NK4* expression [38]. Furthermore, *NK4* expression in the ventral epidermis was shown to be upregulated by BMP treatment and downregulated by pharmacological inhibitors of the BMP pathway [21,36,37]. Because the characteristic phenotype originally described in BMP-inhibited larvae so closely resembled that of our *NK4* CRISPR larvae, we reasoned that *NK4* might act downstream of BMP signaling to pattern the papillae.

To test this, first we showed that overexpression of the BMP antagonist Noggin (*Foxc>Noggin)* also results in a single protruding papilla containing all known papilla cell types (Fig 4A). Second, we found that *Foxc>Noggin* also greatly reduces the expression of the *NK4 > GFP* reporter plasmid in the papillae (Fig 4B). Expression was not completely abolished, but quantification of mean GFP fluorescence (normalized to whole-larva expression of an *Islet>mCherry* reporter) showed that it is significantly reduced upon BMP inhibition (Figs 4C–4E and S4 in S1 File). Interestingly, we observed a complete loss of expression of the the BMP-dependent ventral papilla marker gene *Msx* [21] in Noggin-overexpression larvae, compared to a more modest reduction in *NK4* CRISPR larvae (Fig 4F and 4G). Despite the presence of a single large papilla in in many of these larvae, *Msx* reporter plasmid expression was still observed in the ventral area of the papilla region (Fig 4F). This suggests that, although NK4 functions downstream of BMP signaling to regulate the tripartite organization of the papillae, activation of *Msx* and specification of ventral papilla identity by BMP is mostly independent of NK4.

**Fig 4:**
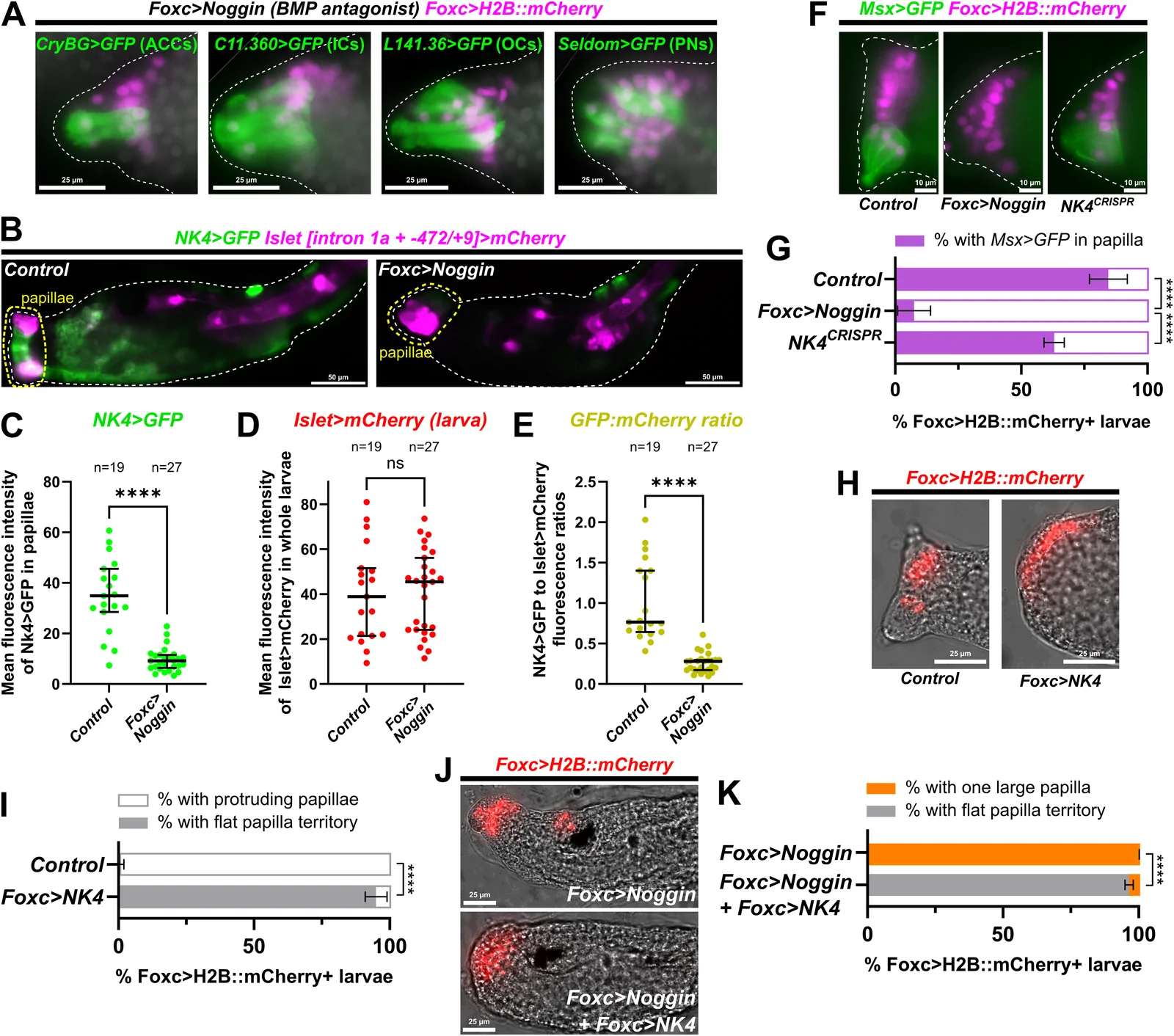
NK4 acts downstream of BMP signaling in the papillae. **A)** Overexpression of the BMP antagonist Noggin in the papilla lineage (using *Foxc>Noggin* plasmid) results in a “*Cyrano”* phenotype similar to *NK4* CRISPR: a single large papilla containing all four papilla cell types (ACCs, ICs, OCs, adn PNs). DAPI counterstain shown in white overlay. All at 17 hpf (St. 27) except *L141.36* reporter expression, which is visible only around 21 hpf (St. 29). **B)** *NK4 >* GFP reporter expression (green) was quantified by measuring mean fluorescence in the papilla territory (yellow dashed area), in negative control and *Foxc>Noggin-*electroporated larvae. Transfection efficacy was normalized using whole-larva expression of *Islet [intron 1a + -472/ + 9]>mCherry* (magenta), which is expressed in papilla and notochord cells. Scale bars = 50 µm. **C)** Comparison of mean fluorescence intensity readouts of *NK4 > GFP* expression in the papilla region (as indicated in panel B), between negative control and *Foxc>Noggin* larvae. Data show significant downregulation of *NK4* reporter expression in the papillae by Noggin overexpression. 5 outliers of fluorescence over 80 were removed from the control dataset. **** p < 0.0001 by two-tailed Mann-Whitney test. Error bars indicate interquartile range. **D)** Comparison of mean fluorescence intensity readouts of *Islet>mCherry* fluorescence in the whole larva, between the same larvae plotted in panel C and shown in panel B. Data show that overexpression of Noggin had no significant effect on Islet expression levels throughout the larva, indicating comparable transfection efficacies and suitable use of whole-larva *Islet* reporter expression to normalize *NK4* reporter activity. p = 0.7404 by two-tailed Mann-Whitney. **E)** Ratio of GFP to mCherry intensity values plotted in panels C and D, showing that *NK4* reporter is significantly downregulated by Noggin overexpression even when normalized by transfection efficacy. p < 0.0001 by two-tailed Mann-Whitney. Outliers with GFP:mCherry ratio >3.0 (three in the control condition) were removed from the entire dataset. Complete data with flagged outliers can be found in S1 Data. Normalization to papilla-specific *Islet>mCherry* expression can be found in S3 Fig in S1 File. **F)** Expression of the *Msx > CD4::GFP* reporter (green) in negative control CRISPR, Noggin overexpression, and *NK4* CRISPR (using *Foxc>Cas9*). **G)** Comparison of percentage of transfected larvae showing *Msx* reporter expression in the ventral papilla region, comparing the conditions shown in panel F. **H)** Overexpression of NK4 using the *Foxc* promoter results in the absence of protruding papillae and flattening of the entire anterior epidermis. **I)** Scoring the percentage of transfected larvae showing the flattened papilla territory, comparing the conditions in panel H. **J)** Overexpression of NK4 in the papilla region can override the Noggin overexpression phenotype, converting a single large papilla into a flattened epidermis. **K)** Scoring the larvae shown in panel J. All scale bars (except in panel B) = 25 µm. All GFP sequences except *Msx > CD4::GFP* are tagged with Unc-76. All scoring experiments were performed in duplicate with n = 100 for all conditions/duplicates (except n = 99 for Foxc>NK4 duplicate 1 in panel I) and averaged, with error bars indicating upper and lower limits. **** p < 0.0001 for both duplicates by Fisher’s exact test.

In contrast to *NK4* CRISPR or BMP inhibition, overexpression of NK4 (*Foxc>NK4*) resulted in the near opposite of the *Cyrano* phenotype: a flat layer of cells where the papillae normally form (Fig 4H and 4I). When combining NK4 overexpression with BMP inhibition (Noggin overexpression), the NK4 overexpression phenotype (flat/no papillae) clearly prevailed (Fig 4J and 4K). Taken together, these data suggest that NK4 acts downstream of BMP signaling in the papilla territory to pattern this region into the three protrusions invariably seen in wild-type larvae.

### NK4 represses *Foxg*

Having established a role for NK4 in patterning the papilla organ downstream of BMP signaling, we next sought to better understand exactly how NK4 achieves this. *NK4* expression has been shown to begin during the neurula stage [38]. By two-color Hybridization Chain Reaction (HCR) mRNA *in situ* hybridization and confocal imaging, we observed overlapping *NK4* and *Foxg* expression at the neurula stage (Figs 5A and S1B in S1 File). A bit later at the mid tailbud stage we see observe *NK4* expression downregulated in the presumptive dorsal papillae, but still expressed in cells of the presumptive ventral papilla and in *Foxg-*negative cells in between the future papillae (Fig 5B). This includes formerly *Foxg+* cells that nonetheless downregulate *Foxg* [27]. Fluorescent signal quantification shows that at this stage *NK4* is expressed at lower levels in more dorsal and lateral regions, especially at the two dorsolateral “spots” of *Foxg* expression corresponding to the two dorsal papillae (Fig 5C).

**Fig 5:**
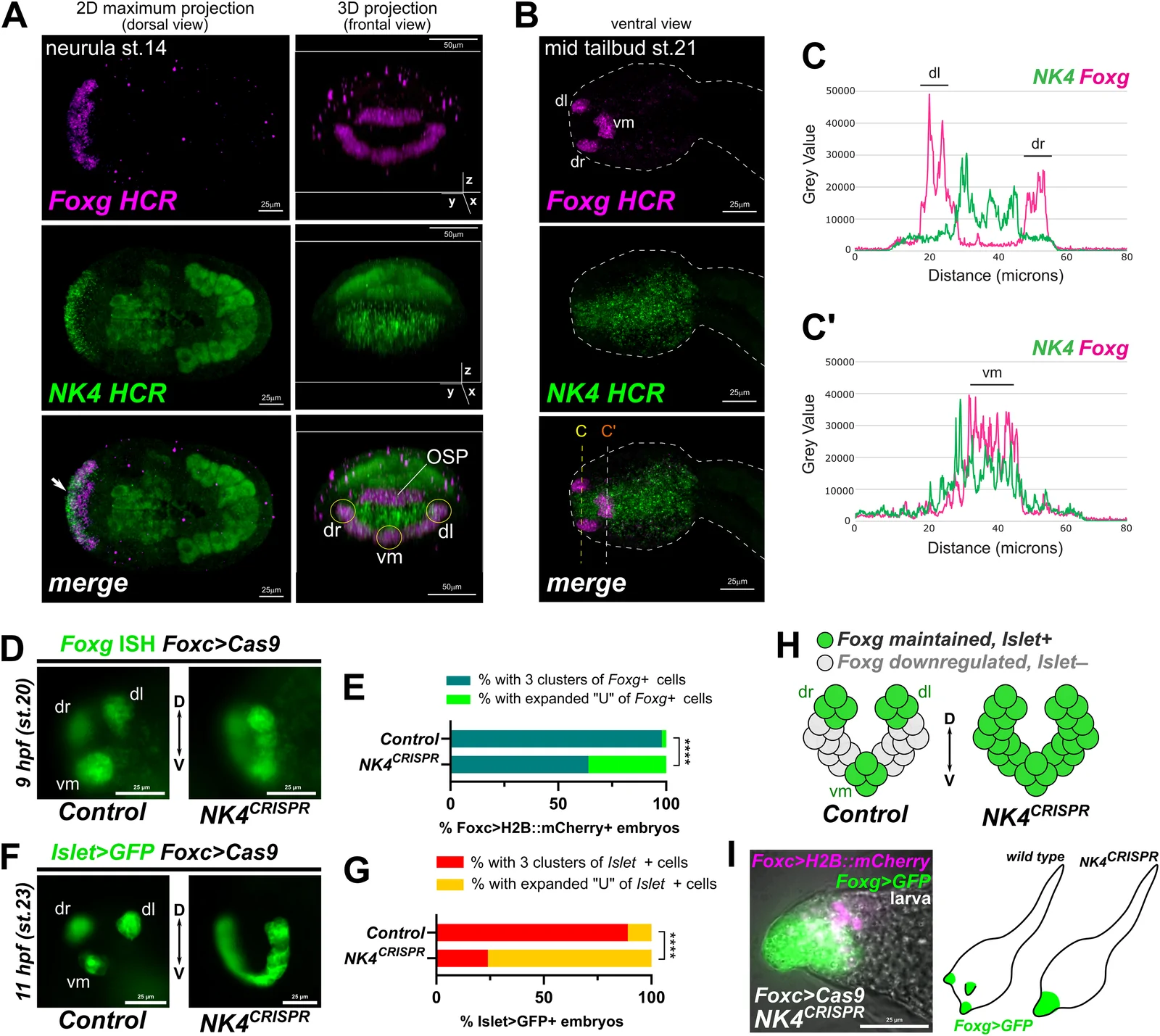
*NK4* CRISPR expands *Foxg* and *Islet* expression. **A)** Whole-mount, two-color fluorescent mRNA *in situ* Hybridization Chain Reaction (HCR) showing the location and extent of *NK4* (green) overlap with *Foxg* (magenta) in the papilla territory (arrow) at neurula stage (Hotta Stage 14). Dorsal view of a 2D maximum projection (left panels) and frontal view of a 3D projection (right panels) shown*.* Quantification of overlap analyzed in S1B Fig in S1 File. Location of cells that will give rise to dorsal right (dr), dorsal left (dl) and ventral medial (vm) papillae, and oral siphon placode (OSP) are indicated. Embryo slightly tilted downward in the frontal projection to avoid obscuring the papillae with signal from autofluorescent tail muscles. **B)** HCR showing location of *Foxg* and *NK4* overlap at the mid-tailbud stage (~9 hours post-fertilization, or hpf, Hotta stage 21). **C)** Plot profiles showing mean fluorescence intensities (grey values) for *NK4* (green) and *Foxg* (magenta) along yellow dashed line in panel B. The location of cells that will give rise to the right and left dorsal papillae are indicated, showing lack of overlap at future dorsal papillae. **C’)** Similar quantification plot as in panel C, but along orange dashed line. Location of cells that give rise to the ventral medial papilla indicated, showing extensive overlap here. **D)** *In situ* hybridization of *Foxg* at 9 hpf (mid tailbud), showing expansion from three spots to a “U” shape swath of cells upon *NK4* CRISPR, compared to negative control CRISPR embryos. **E)** Scoring percentage of transfected embryos showing expanded *Foxg* expression as shown in panel B. n = 100 for both conditions. **F)** Expansion of *Islet>GFP* reporter expression at 11 hpf (late tailbud) upon *NK4* CRISPR. Control embryos were instead electroporated with the *Defcab.47* sgRNA, targeting a gene that is not expressed in the papillae (see materials and methods for details). **G)** Scoring of transfected embryos showing expanded *Islet* reporter expression as seen in panel D. For control, n = 80, and for CRISPR n = 97. **H)** Summary diagram explaining our interpretation of expanded *Foxg* and *Islet* expression in *NK4* CRISPR embryos. **I)** Left: *NK4* CRISPR larva showing *Foxg* reporter expression (green) in the resulting single large papilla. Right: schematic diagram explaining the expanded *Foxg* and single papilla phenotype in *NK4* CRISPR larvae. dr: dorsal right; dl: dorsal left; vm: ventral medial; D: dorsal; V: ventral. All scale bars = 25 µm except for 3D projections in panel A (50 µm). All GFP sequences are tagged with Unc-76. **** p < 0.0001 by Fisher’s exact test.

Due to the fact that Foxg promotes Islet expression, and that both are required for the formation of the three protrusions later on [20,27,28], we hypothesized that NK4 might downregulate *Foxg* in cells between and surrounding the three *Foxg + /Islet+* cell clusters. Indeed, we found that *NK4* CRISPR results in ectopic *Foxg* expression, observed by *in situ* at the mid-tailbud stage (Fig 5D and 5E). Instead of the three characteristic *Foxg+* cell clusters, we observe a “U”-shaped stripe of *Foxg* expression. This phenocopied the expansion of *Foxg* in BMP-inhibited larvae previously reported [20,21]. We further observed that NK4 CRISPR similarly expands Islet reporter expression from three dots to a U-shaped stripe, like the expansion of Islet observed under BMP inhibition (Fig 5F and 5G) [21]. It is thought that the expansion of these three *Foxg + /Islet+* clusters into a single contiguous group (Fig 5H) results in the enlarged protrusion seen in BMP-knockdown larvae [20,21]. Indeed, *Foxg>GFP* reporter expression was observed throughout the single large papillae of *NK4* CRISPR larvae (Fig 5I).

Conversely, NK4 overexpression (*Foxc>NK4*) suppressed *Foxg* and downstream papilla cell type-specific reporter expression (Figs 6A–6C and S4A in S1 File). While *Foxg>GFP* was still visible in a substantial portion of NK4-overexpressing larvae (44%, Fig 6B), it was significantly reduced in fluorescence intensity (Fig 6C). When we overexpressed NK4 (*Foxc>NK4)* and Foxg (*Foxg>Foxg*) together in the same embryos, a majority of larvae developed a flat papilla territory, more closely resembling the phenotype elicited by *Foxc>NK4* alone (Figs 6D and S4B in S1 File). Taken together, our data suggest that NK4 is necessary and sufficient to repress Foxg and the specification of protruding papillae. Curiously, *Foxc>NK4* does not appear to suppress the expression of the *Islet* reporter (S4C Fig in S1 File), suggesting a more complex regulatory logic for this particular gene. On the other hand, earlier overexpression of NK4 using the *Friend of GATA (FOG)* promoter (before the specification of the papilla territory) resulted in severely malformed larvae lacking discernible papillae (S4D Fig in S1 File).

**Fig 6:**
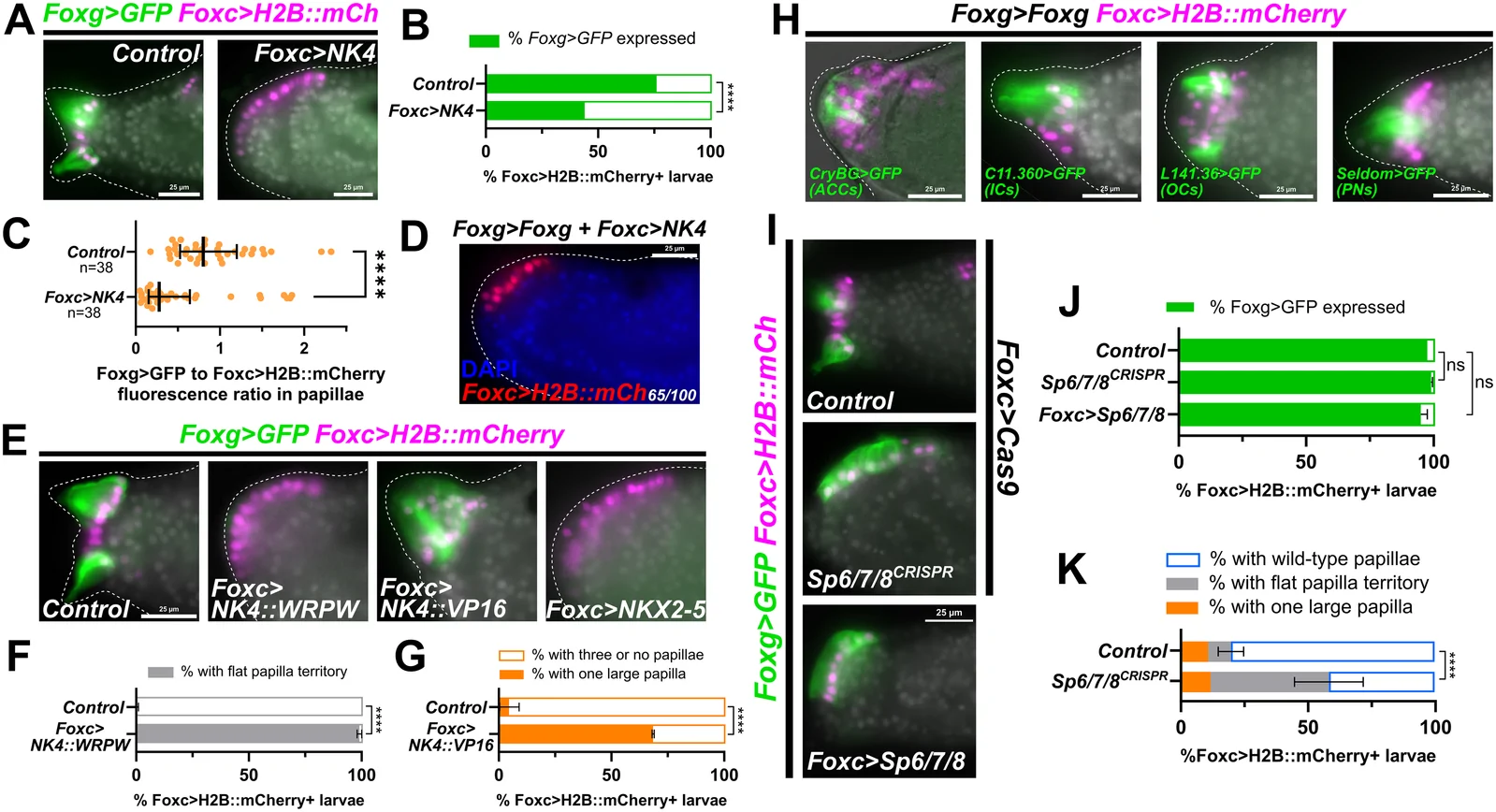
NK4 acts as a transcriptional repressor to suppress *Foxg* expression in the papillae. **A)** Overexpression of NK4 suppresses *Foxg* reporter expression (green) in the flattened papilla region. **B)** Scoring of *Foxg>GFP* expression frequency comparing *Foxc>NK4* and negative control larvae. **C)** Quantification of *Foxg>GFP* and *Foxc>H2B::mCherry* relative expression levels in the papillae, comparing NK4 overexpression to negative control condition as represented in panel A. **** p < 0.0001 by two-tailed Mann-Whitney test. **D)** Single large papilla elicited by Foxg overexpression can be suppressed by co-overexpression of NK4 (in 65 out of 100 larvae in duplicate 1, 82/100 in duplicate 2, see scoring plot in S4B Fig in S1 File). **E)** Overexpression of NK4::WRPW, in which the C-terminus of NK4 (C-terminal to the homeodomain) is replaced with the WRPW Groucho co-repressor recruitment domain, phenocopies full-length NK4 overexpression. Replacing the C-terminus instead with a VP16 transactivation domain (NK4::VP16) resulted in a single large papilla with robust *Foxg>GFP* expression, similar to *NK4* loss-of-function. Overexpression of flag-tagged human NKX2-5 results in flat papilla, phenocopying *Ciona* NK4 overexpression. Scoring data for NKX2-5 overexpression is shown in S5A and S5B Fig in S1 File). **F)** Scoring the percentage of transfected larvae with either normal, protruding papillae or flat/no papillae, comparing NK4::WRPW overexpression to negative control larvae. **G)** Scoring the percentage of transfected larvae showing a single large papilla, comparing NK4::VP16-ovexpressing larvae to negative control larvae. NK4::VP16 also resulted in 30% larvae with no protruding papillae (see S1 Data for scoring details). **H)** Overexpression of *Foxg* by electroporating the *Foxg>Foxg* plasmid results in a single large papilla containing all four cell types, similar to that seen in *NK4* CRISPR and Noggin overexpression. OCs imaged at 21 hpf (St. 29) due to later onset of *L141.36* expression. ACC experiment conducted in a *Foxg* CRISPR background and imaged at 19 hpf (St. 28) as part of a rescue experiment described previously [15], see S1 Text for details. **I)** CRISPR knockout of *Sp6/7/8* does not phenocopy BMP inhibition or *NK4* CRISPR. While *Foxg>GFP* is still expressed, the papilla territory is most frequently flat. Sp6/7/8 overexpression also does not phenocopy NK4 overexpression, as *Foxg>GFP* expression is not repressed. **J)** Scoring for *Foxg>GFP* expression in conditions shown in panel I. **K)** Scoring for flat, wild type, or a single large papilla, comparing *Sp6/7/8* CRISPR to negative control larvae. All larvae counterstained with DAPI (white or blue overlay) except *CryBG/*ACC image in panel C. All GFP sequences fused to Unc-76. All scale bars = 25 µm. mCh: mCherry. All scoring experiments had n = 100 for all conditions/duplicates. For those performed in duplicate, frequencies were averaged and error bars indicate upper and lower limits. **** p < 0.0001 for both duplicates by Fisher’s exact test. ns = not significant.

NK4 and its orthologs in other species have been described as both transcriptional repressors and activators [39,40]. To probe whether NK4 is acting as a repressor or activator in the papilla territory, we replaced the C-terminus of NK4 with either a WRPW repressor domain [41] or a VP16 transactivation domain [42], and expressed either throughout the papilla territory using the *Foxc* promoter. The overexpression of NK4::WRPW phenocopied the full-length NK4, resulting in downregulation of *Foxg>GFP* and a flat papilla territory (Fig 6E and 6F). Strikingly, the effects of overexpressing NK4::VP16 appeared similar to the *NK4* CRISPR phenotype, resulting in a single, large *Foxg+* papilla (Fig 6E and 6G). We also found that overexpression of all three human NK4 genes (NKX2–3, NKX2–5, and NKX2–6) closely resembled the overexpression of *Ciona* NK4, repressing *Foxg* reporter activity and flattening the papilla region (Figs 6E and S5 in S1 File). Taken together, these data suggest that *Ciona* NK4 normally functions as a transcriptional repressor of *Foxg* expression during papilla morphogenesis, and that this repressor function may be highly conserved in vertebrate NK4 orthologs.

It was previously shown that Foxg overexpression results in a single large papilla protrusion [27]. To further test whether impaired refinement/downregulation of *Foxg* likely underlies the phenotype seen in BMP inhibition and *NK4* CRISPR conditions, we electroporated a *Foxg>Foxg* plasmid to overexpress Foxg override the normal downregulation of *Foxg*. Indeed, the *Foxg>Foxg* plasmid results in a single enlarged papilla with all four major papilla cell types present, similar to both BMP inhibition and *NK4* CRISPR (Fig 6H). Based on these results, we conclude that the single large protruding papilla seen with *NK4* CRISPR is due to impaired downregulation of *Foxg* in cells outside the three cell clusters that normally give rise to the three papillae.

It was previously proposed that the transcription factor Sp6/7/8 (also known as Zf220) could downregulate *Foxg,* refining its expression to the three papillae. To test this, we knocked out *Sp6/7/8* in the papilla territory using previously published sgRNAs, but this did not phenocopy BMP inhibition or *NK4* CRISPR, resulting instead most often in a flattened papilla region even though *Foxg* reporter was still expressed (Fig 6I and 6K). Unexpectedly, overexpression of Sp6/7/8 (*Foxc>Sp6/7/8)* did not repress the *Foxg* reporter (Fig 6I and 6J). Based on these results we propose that NK4 is the key transcription factor regulating *Foxg* expression and the number of protruding papillae downstream of BMP, although *Sp6/7/8* perturbation also affects papilla morphogenesis in unexpected ways.

### Chordin restricts NK4 expression and allows for dorsal papilla formation

Having found that NK4 can repress *Foxg*, we sought to understand how the Foxg+ papilla protrusions are allowed to form. By HCR *in situ* hybridization at the mid-tailbud stage, *NK4* expression is weaker in the two dorsal papillae and does not extend more dorsally (Fig 5A–5C). We hypothesized that *NK4* might be downregulated at the very dorsal and lateral edges of the papilla territory, enough to allow for sustained *Foxg* expression in the cells that eventually give rise to the two dorsal protrusions. Notably, the BMP antagonist Chordin is expressed in the lateral border of the neural plate, immediately posterior to the future dorsal papillae [20,38]. As the neural plate folds to form the neural tube and the papilla territory comes to occupy its position at the anterior end of the larval head, the Chordin+ cells intercalate at the dorsal midline and become situated immediately posterior to the dorsal papillae (Fig 7A). We hypothesized that Chordin might be able to limit *NK4* expression more dorsally/laterally/posteriorly through its ability to antagonize BMP signaling. Indeed, when we overexpressed Chordin (*Foxc>Chordin*), we obtained a similar effect as seen with Noggin overexpression, in which *NK4* reporter was downregulated and a single papilla protrusion formed (Figs 7B, 7C and S6 in S1 File). In contrast, CRISPR/Cas9-mediated knockout of *Chordin* in the ectoderm significantly upregulated *NK4* and expanded *Msx* reporter expression in the papilla territory, suggesting more elevated BMP signaling (Figs 7D, 7E, S7 and S8 in S1 File). By quantifying normalized mean *NK4 > GFP* fluorescence, we demonstrated that its expression in negative control larvae is indeed lower in the dorsal half of the papilla territory than in the ventral (Fig 7D and 7E). CRISPR knockout of *Chordin* increased *NK4* reporter expression, especially in the dorsal half of the papilla territory, closest to the source of Chordin (Fig 7D and 7E). As expected, *Chordin* CRISPR suppressed dorsal *Foxg* reporter expression and dorsal papilla formation (Fig 7F and 7G). Based on these data, we propose that Chordin limits the reach of BMP-activated *NK4* expression on the dorsolateral edges of the papilla territory, allowing for proper *Foxg* expression there and specification of the dorsal papillae.

**Fig 7:**
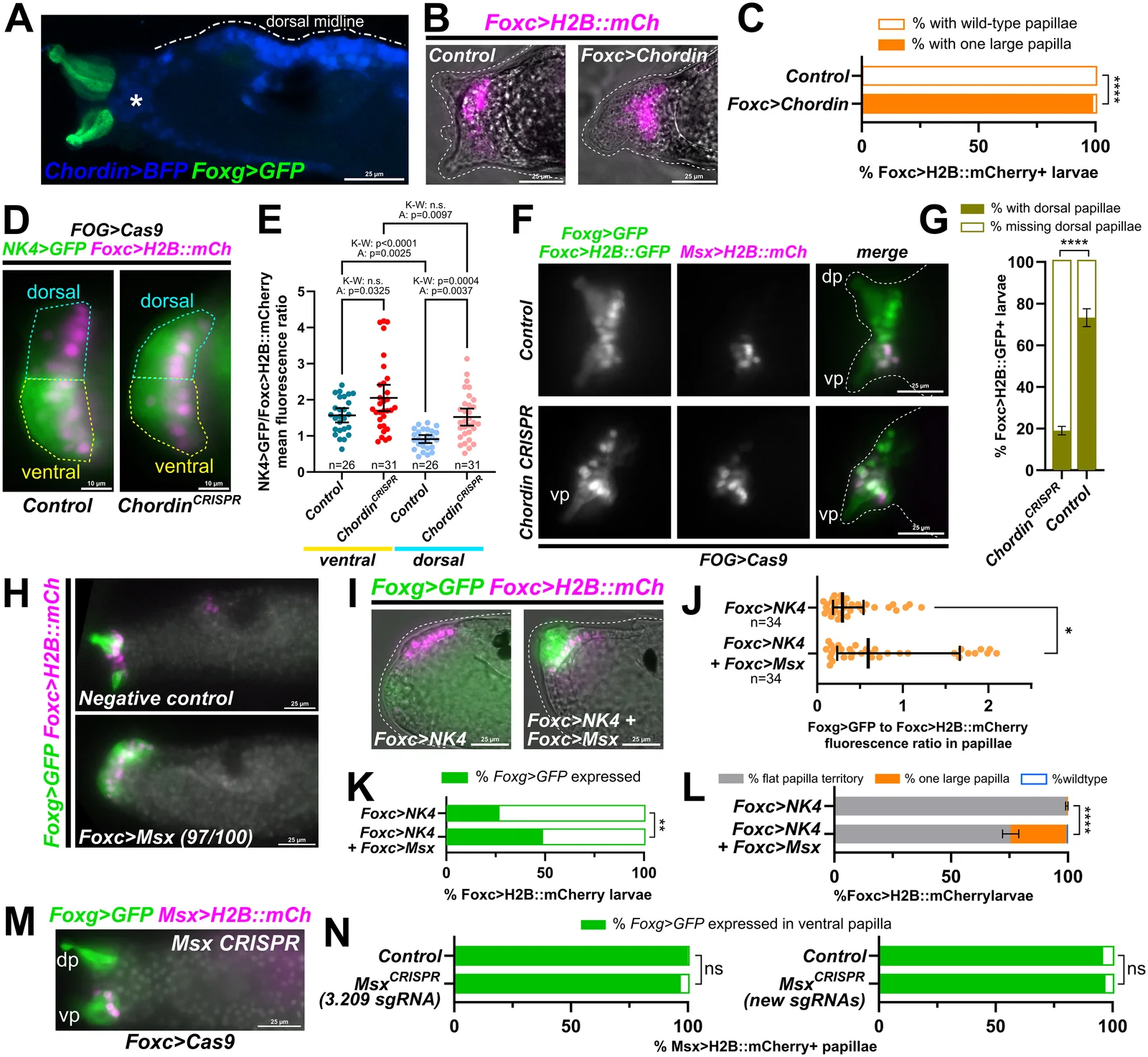
Regulation of NK4 expression and activity by Msx and Chordin. **A)** A larva co-electroporated with *Chordin>TagBFP* (blue) and *Foxg>GFP* (green) reporter plasmids. *Chordin* is expressed in lateral neural plate border cells that intercalate to give rise to dorsal midline cells posterior to the dorsal papillae [20]. Asterisk denotes migratory mesenchyme cells, which express *Chordin* at the tailbud stage [38]. **B)** Overexpression of Chordin in the papilla territory phenocopies Noggin overexpression, suggesting it antagonizes BMP signaling as predicted. **C)** Scoring the percentage of transfected larvae showing either wild-type three normal papillae or a single large papilla, comparing Chordin overexpression and negative control larvae, as in panel E. **** p < 0.0001 per Fisher’s exact test, n = 100 for both conditions. **D)** Examples of the papilla territory divided into ventral and dorsal halves and imaged for *NK4 > GFP* (green) and *Foxc>H2B::mCherry* (magenta) quantification, divided into dorsal and ventral halves, to compare *NK4 > GFP* expression in negative control and *Chordin* CRISPR larvae. Scale bars = 10 µm. **E)** Ratios of GFP to mCherry mean fluorescence intensities were calculated for ventral and dorsal halves, across control and *Chordin* CRISPR conditions as shown in panel G. One-way ANOVA and Kruskal-Wallis tests were performed in parallel as the data for ventral papillae in *Chordin* CRISPR was not normally distributed on account of a few outliers. See text for discussion of possible interpretations. n.s.: not significant (p > 0.05). **F)** *Chordin* CRISPR can eliminate the dorsal papillae, which are labeled by *Foxg>GFP* (green cytoplasm) but are negative for *Msx > H2B::mCherry* (magenta). dp: dorsal papilla(e), vp: ventral papilla. **G)** Scoring of larvae expressing *Foxc>H2B::GFP+* (green nuclei), showing presence or absence of dorsal papillae in control or *Chordin* CRISPR conditions as seen in panel F. Experiment performed and averaged between duplicates, with n = 100 for all conditions/duplicates except CRISPR duplicate 1 (n = 200) and errors bars indicating upper/lower range. **** p < 0.0001 for both duplicates, per Fisher’s exact test. **H)** Overexpression of the ventral papilla-specific regulator Msx promotes GFP results in a single large, though blunt papilla (observed in 97 out of 100 larvae in duplicate 1, 99/100 in duplicate 2). **I)** Msx can partially rescue the NK4 overexpression-induced loss of *Foxg* reporter expression and papilla formation. **J)** Quantification of GFP:mCherry fluorescence ratios in the papillae of larvae, in a replicate of the experiment shown in panel I, demonstrating that overexpression of Msx rescues *Foxg>GFP* expression in NK4 overexpression condition. * p = 0.0158 by two-tailed Mann-Whitney test. **K)** Scoring the percentage of transfected larvae showing *Foxg>GFP* reporter expression, comparing NK4 and Msx co-overexpression vs. NK4 overexpression alone, as shown in panel I. ** p = 0.0021 by Fisher’s exact test. **L)** Scoring the percentage of larvae showing a flat papilla territory, a single large papilla, or the normal three papillae, comparing the conditions in panel I. **** p < 0.0001 for both duplicates, per Fisher’s exact test looking at flat and one large papilla categories. n = 100 for all conditions/duplicates except NK4 + Msx duplicate 1 (n = 102). **M)** *Msx* CRISPR does not affect *Foxg* expression or ventral papilla specification (shown here using new *Msx* sgRNAs). **N)** Scoring of *Foxg* expression in the ventral papillae of *Msx* CRISPR, showing no loss with either a published sgRNA (3.209) or new sgRNAs. n = 100 for each condition/duplicate. ns = not significant by Fisher’s exact test. All GFP sequences fused to Unc-76 except otherwise noted. All scale bars = 25 µm unless otherwise specified.

### Msx can dampen the repression of *Foxg* but is not required for ventral papilla formation

While Chordin is required for dorsal papilla formation, we next asked how the ventral medial protrusion forms. This protrusion arises from an area with high levels of BMP signaling [21] and NK4 expression. Indeed, by *in situ* hybridization, strong *NK4* and *Foxg* expression overlap in the ventral medial papilla (Fig 5A), which is known to express *Msx*. We therefore hypothesized that Msx might somehow interfere with NK4 function and/or activate *Foxg.* Consistent with this, overexpression of Msx (*Foxc>Msx)* results in a single large, if somewhat blunt, contiguous *Foxg+* papilla territory (Fig 7H). We next co-electroporated equal amounts of *Foxc>Msx* and *Foxc>NK4*, to see if the former could antagonize the latter’s repressive effect on *Foxg* reporter expression and papilla formation. Indeed, co-electroporating *Foxc>Msx* and *Foxc>NK4* partially rescued *Foxg* reporter expression (Fig 7I–7K), resulting even in a large single protrusion in ~25% of cases (Fig 7L).

While the overexpression experiments suggested that Msx can promote *Foxg* expression in NK4 + cells, this effect was relatively modest, leading us to hypothesize that there may be additional factors contributing to the de-repression of *Foxg* in the ventral papilla. To test whether *Msx* is necessary for ventral papilla formation, we used CRISPR to knock it out in the papilla territory. Neither a previously published sgRNA targeting *Msx* [43]*,* nor newly designed and validated sgRNAs resulted in loss of the ventral papilla (Fig 7M, 7N and S9A in S1 File). These results suggest that *Msx* is not required for *Foxg* expression and specification of the ventral papilla.

Interestingly, at the late tailbud stage we observed a “hole” in the *NK4* expression domain by *in situ* HCR, right at the location of the future ventral papilla (S1C Fig in S1 File). This suggests that *NK4* might also be downregulated here, contributing to ventral papilla formation. However, when we overexpressed Msx throughout the papilla territory, *NK4* reporter expression frequency was not reduced (S9B–S9D Fig in S1 File). This suggests that a different, unidentified factor may downregulate *NK4* specifically in the ventral papilla, possibly downstream of Foxg.

## Discussion

Here we present evidence for the involvement of NK4 as a key mediator of BMP signaling in patterning the sensory-adhesive papillae of tunicate larvae. It was previously proposed that BMP is required in the ventral papilla for the expression of an unknown, secreted factor that activates the expression of Sp6/7/8 (also known as Zfp220)*,* which in turn was proposed to repress *Foxg* in interleaving cells to produce the tripartite organization of the papilla organ [20,21,27]. This was partially based on lack of clear phosphoSMAD immunostaining outside the most ventral part of the papilla territory [21]. Here we show that the missing factor may be NK4, and that *Sp6/7/8* need not be invoked. Instead of being secreted, NK4 is predicted to act cell-autonomously. We suggest that NK4 expression is activated by low levels of BMP signaling, beyond the limits of robust phosphoSMAD detection, which is consistent with our data and that of other studies tightly linking *NK4* expression to BMP signaling [21,35–37].

We propose that the patterning of the papillae can be partially explained by a classical morphogen gradient (BMP) with *NK4* as its primary target (Fig 8). We show that NK4 is a transcriptional repressor that is necessary and sufficient to repress *Foxg* in the papillae, and that its expression is determined by a gradient of BMP activity in the papilla territory. BMP signaling appears to be limited laterally (early) and dorsally (late) by Chordin, with the gradient axes shifting as morphogenesis results in lateral neural plate border cells becoming situated more dorsally, while medial cells end up positioned more ventrally. Loss of BMP or NK4 results in ectopic *Foxg* activation in intervening cells that normally downregulate *Foxg,* promoting the formation of a single, large papilla (the “*Cyrano*” phenotype). Future work will be needed to confirm some of these predictions, and to ascertain which regulatory connections are direct or indirect*,* but this model is consistent with the data presented in the current study. It will also be interesting to investigate the roles of BMP and NK4 in tunicate species whose larvae do not have an overtly tripartite morphology. Is the expression of NK4 in the papilla territory of these species altered? Or are there still underlying, cryptic tripartite papilla cell type organization or gene expression patterns? Interestingly, tripartite expression of another regulatory gene, *Pitx,* follows a similar refinement from a “U”-shaped stripe to three dots in the tripartite papillae of the distantly related *Halocynthia roretzi* [26], which could suggest that a BMP-NK4 papilla patterning mechanism may have been present in the last common ancestor of all extant tunicates.

**Fig 8:**
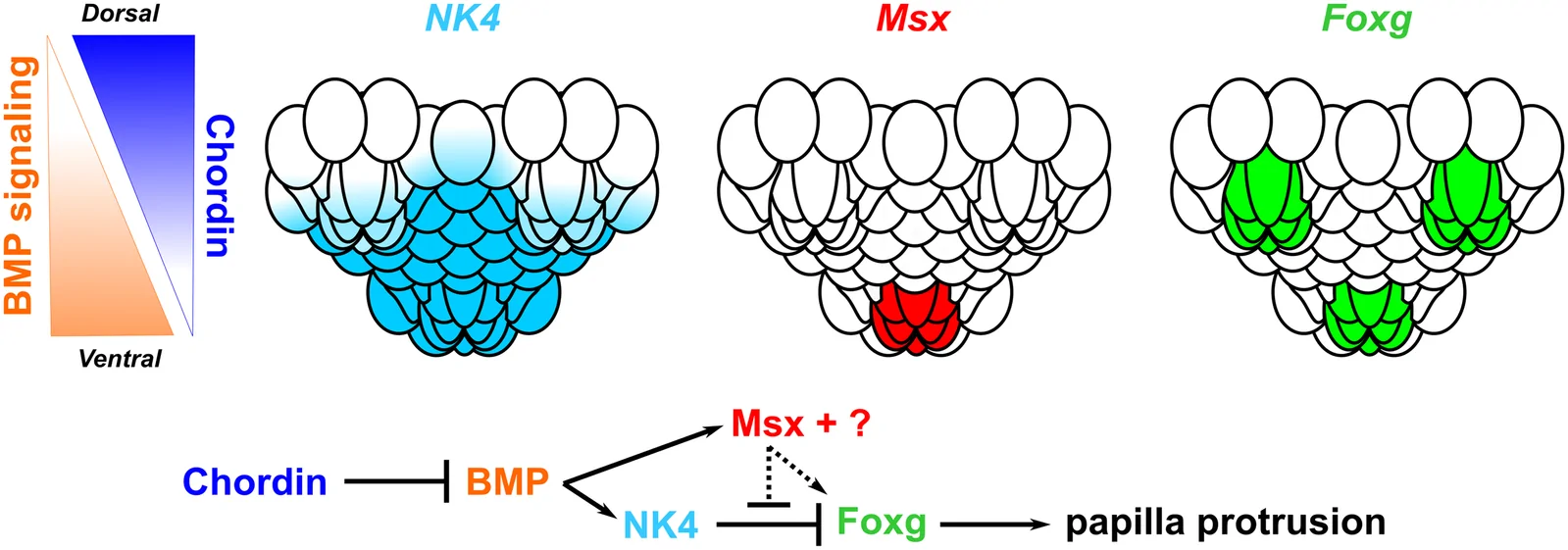
The proposed role of NK4 downstream of BMP in the papillae. A diagram summarizing the main points of the current work. Top left: A ventral-to-dorsal gradient of BMP signaling is shaped in part by an opposing, dorsal-to-ventral gradient of the BMP antagonist Chordin. Top right: expression domains of *NK4, Msx,* and *Foxg* in the papilla territory (dorsal at top). According to our proposed model, NK4 downregulates *Foxg* in certain intervening cells, but Msx may interfere with NK4 function and/or help promote *Foxg* expression in the ventral-most cells, together with other factor(s) yet to be identified. Bottom: proposed regulatory network in which NK4 downregulates *Foxg* downstream of BMP signaling.

In the ventral-most part of the papilla territory, where BMP signaling is at its highest, we propose that Msx can interfere with the ability of NK4 to repress Foxg, which might contribute to the formation of the ventral papilla protrusion. However, our CRISPR results suggest that Msx is not required for ventral papilla *Foxg* expression. One possibility is that Msx acts in parallel with additional ventral factors to promote *Foxg* expression. The downregulation of *NK4* in the ventral papilla observed later in development (S1A and S1C Fig in S1 File) may be in part due to such parallel, yet-to-be-identified factors (summarized in an alternative model presented in S10 Fig in S1 File). Indeed, Msx co-expression only partially alleviated the repressive effect of NK4. Alternatively, it is possible that Msx can promote the maintenance of Foxg expression in the ventral papilla, but is not required for its initial activation there.

While our data strongly support a role for Chordin in antagonizing *NK4* expression in the dorsal papillae it is still unclear if this activity extends to more ventral areas. In our *Chordin* CRISPR larvae, different statistical tests were split on whether the increase in *NK4* reporter expression was statistically significant in the ventral half of the papilla territory, compared to that in negative control larvae (Fig 7E). Similarly, statistical tests were split on whether knocking out *Chordin* results in more uniform *NK4* reporter expression along the dorsal-ventral axis of the papillae. Our measurements were likely hindered by the obvious morphological defects in the dorsal papillae elicited by *Chordin* CRISPR (Fig 7F). However, we also observed a significant expansion of *Msx* reporter expression (another BMP target) in the papilla territories of our *Chordin* CRISPR larvae (S8B Fig in S1 File), suggesting that Chordin likely influences the ventral papilla as well.

It is unclear why *Foxg* is normally downregulated at the very dorsolateral edges of the papilla territory, where presumably NK4 should not be present to repress it. Here, regulation of *Foxg* may be independent of BMP and NK4, as suggested by the lower sensitivity of *Sp6/7/8* expression to BMP inhibition in these cells as previously reported [20]. Similarly, NK4 might not be needed to repress *Foxg* in the very center of the papilla territory, which is derived from *Emx+* cells that never activate *Foxg* to begin with (Fig 1) [27].

One outstanding question is how to reconcile this model with data supporting alternative models. For instance, the MEK/ERK pathway (presumably downstream of FGF) was previously suggested to limit *Islet* expression to the three spots/protrusions, with pharmacological inhibition of MEK at the late neurula stage (stage 16) resulting in an expanded domain of *Islet* expression similar to that seen with BMP inhibition or *NK4* CRISPR [28]. MEK inhibition also suppresses the specification of Islet-negative cell types, such as outer collocytes and papilla neurons [15]. It is possible that MEK/ERK signaling might provide a temporal, not spatial, restriction on *Islet* expression. We know that MEK/ERK signaling is active in the lineage at a slightly earlier stage to promote *Foxg* expression in papilla precursors [27]. If MEK/ERK signaling can repress *Islet* expression at this earlier stage (when its upstream activator, Foxg, is already present), it would explain why *Islet* is normally not expressed in a U-shaped territory like we see for *Foxg* early on*.* In other words, this might constitute a time-delay mechanism to ensure that *Islet* is only expressed in three dots, after *Foxg* expression has been refined. Future work would be needed to investigate this, with carefully timed *in situ* hybridizations to see if *Islet* is indeed precociously activated upon MEK inhibition.

Another unresolved question is the exact role(s) of Sp6/7/8 in patterning the papillae. Previously we have shown that Sp6/7/8 is required for the proper specification of collocytes in the papillae, but this appears to complement, not repress, Foxg-mediated gene expression [15]. Furthermore, Sp6/7/8 is expressed in papilla neuron progenitors, but is not required for neurogenesis [15]. Neither BMP inhibition nor *NK4* CRISPR appear to prevent the formation of collocytes or papilla neurons (Figs 3A, 3B and 4A). Similarly, *Sp6/7/8* CRISPR does not phenocopy BMP inhibition or *NK4* CRISPR, while overexpression of Sp6/7/8 (*Foxc>Sp6/7/8)* did not repress *Foxg* reporter (Fig 6J–6L). Hence, more work will be needed to study the precise regulatory relationship between Sp6/7/8 and Foxg.

Finally, previous work has pointed out the evolutionary parallels and connections between anterior neural border specification in tunicates and vertebrates, especially highlighting the role of BMP signaling [17,20,21]. Of note, the cement gland of frogs arises from an equivalent anterior ectodermal territory and its development is also regulated by BMP [44]. However, it is unclear if NK4 orthologs in vertebrates play a similar role in BMP-dependent ectoderm patterning as we show here in *Ciona.* While *Ciona* and other tunicates have only one NK4-family protein, in vertebrates these have duplicated and sub-functionalized into three ohnologs in most species: *Nkx2.3, Nkx2.5,* and *Nkx2.6/7* (S11 Fig in S1 File) [45]. We show here that the overexpression of at least one of those, human NKX2–5, can phenocopy *Ciona* NK4 overexpression (Figs 6F and S5 in S1 File). In zebrafish, *Nkx2.3* and *Nkx2.7* are activated in pharyngeal endoderm by BMP [46], which may point to an ancient BMP-NK4 regulatory mechanism for patterning of head structures. This may be further connected to a more ancient patterning function for BMP [47], though a conserved role for NK4-family factors in this is unclear. Interestingly, a requirement for *Nkx2.3* in orofacial ectoderm, specifically in salivary gland and ameloblast (tooth) development, has been shown in mouse [48–50]. BMP signaling is also critical for salivary gland [51] and tooth development [52,53], and has been shown to be upregulated in the dental epithelium by Nkx2.3 [54]. However, whether Nkx2.3 is also downstream of BMP signaling in the orofacial ectoderm remains to be seen, to our knowledge. We have previously expressed support for a deeper evolutionary link between the papillae of tunicate larvae and various vertebrate surface ectodermal organs like secretory glands and teeth [32]. Our findings might suggest an ancient role for NK4 in mediating BMP patterning of ectodermal organs in the last common ancestor of vertebrates and tunicates.

## Materials and methods

### Ciona handling and electroporation

Gravid adult specimens of *Ciona robusta* (*intestinalis* Type A) were collected and shipped by Marinus Scientific (Lakewood, CA) or M-REP (Carlsbad, CA). Gametes were isolated from adults and fertilized *in vitro* and dechorionated as previously described [55]. Electroporation of dechorionated, synchronized zygotes was carried out as previously described [56]. Plasmid DNA electroporation amounts were calculated and listed as total micrograms per 100 µl, for a final electroporation volume of 700 µl (including D-mannitol solution and sea water). DNA sequences and amounts are listed in the Supplemental Sequences file (S1 Text). Embryos, larvae, and juveniles were reared at 20°C, and staged according to hours post-fertilization (hpf) and Hotta Staging [57]. To monitor metamorphosis, larvae were transferred at 17 hpf (Hotta Stage 27) to a new agarose-coated 10 cm petri dish with 400 µl of 100X penicillin-streptomycin stock solution (Gibco) added to each dish. Juveniles were collected at 44 hpf (stage ~35) and fixed according to the same protocol as for larvae. Larvae were collected and fixed at 17 hpf, unless otherwise noted.

### Previously published non-coding sequences used

Various published non-coding sequences from *Ciona* were adapted for use in this study, among them, *Friend of GATA (FOG)* [30], *Foxc* [25], *CryBG* [58], *Seldom (KH.C4.78)* [15], *KH.C11.360* [15], *KH.L141.36* [15], *Islet* [28], *Foxg* [59], *Msx* [60], and *Chordin* [17].

### Immunofluorescence, HCR, and imaging

Larvae and embryos were fixed as previously described for traditional whole-mount mRNA *in situ* hybridization [61], or for immunofluorescence or fluorescent protein imaging [62]. For *In situ* hybridization chain reaction (HCR), embryos and larvae were fixed and treated as described in [63] using HCR RNA-FISH v3.0 probes, amplifiers, and buffers obtained from Molecular Instruments (Los Angeles, USA). *Foxg* probe for traditional *in situ* was previously published [32]. Acetylated tubulin antibody staining was performed modified from a published protocol [64]. Briefly, larvae were fixed by direct addition of 16% paraformaldehyde (250 µl for a 10 cm dish), then washed four times in PBS + 0.1% Tween-20. Larvae were incubated at 4°C overnight in mouse anti-acetylated primary antibody (Sigma-Aldrich catalog# MABT868) diluted 1:200 in PBS + 1% BSA, then washed four times in PBT. Secondary antibody (donkey anti-mouse AlexaFluor-488, ThemoFisher catalog# A21202) was incubated at 1:400 in PBS + 1% BSA again overnight at 4°C. Final washes were performed as before, prior to incubating with DAPI and mounting. Images were acquired on Leica DMIL LED or DMI8 inverted compound epifluorescence microscopes, or a Zeiss LSM 900 laser-scanning confocal microscope as indicated. Images acquired on Leica microscopes were processed and exported using LASX software, while confocal images were processed using ImageJ2/FIJI [65] or the ZEN (version 3.10) software suite. For scoring, only embryos/larvae expressing *Foxc>H2B::mCherry* in the papillae were scored, unless otherwise specifically noted. Fisher’s exact test was performed using the Prism GraphPad calculator online: https://www.graphpad.com/quickcalcs/contingency1/

### NK4 reporter fluorescence quantification and normalization

Fixed, slide-mounted larvae were imaged on a Leica DMI8 inverted compound epifluorescence microscope using the same illumination, exposure, and gain settings between larvae and conditions for a given experiment (though settings changed depending on whether it was GFP or mCherry fluorescence being imaged. For the Noggin overexpression experiment, the 20X objective (HC PL FLUOTAR L, 0.40 N.A., 0–2 mm correction collar) was used to image both the papillae and most of the larva (excluding the tail tip). For the Chordin CRISPR and Chordin overexpression experiments, the 40X objective (HC PL APO, 0.95 N.A., 0.11-0.23 mm correction collar) was used to image mostly the head including the papillae. After image acquisition, regions of interest (ROIs) were drawn in Leica LASX software, and mean fluorescence pixel intensity values exported for further analysis and plotting. For the Noggin overexpression experiment, an ROI around the papilla region and another ROI around the whole larva was drawn for each individual. For the Chordin CRISPR experiment, one ROI was drawn around the ventral half of the papilla region, and another around the dorsal half of the papilla region, for each individual. Raw mean pixel intensity measurements and calculated GFP/mCherry ratios can be found in S1 Data.

### Tissue-specific CRISPR/Cas9-mediated mutagenesis in F0

CRISPR/Cas9-mediated mutagenesis in electroporated embryos was carried out as previously described [66]. The original *NK4*-targeting sgRNA (*NK4.3*) was previously published and validated by in-house Illumina amplicon sequencing [34]. A negative control (“Control”) sgRNA targeting no *Ciona* sequence was previously published [67]. An additional sgRNA (*Defcab.*47) targeting the *Defcab* gene [68,69], which is not expressed in the papillae, was similarly used as a negative control in one experiment (Fig 5F and 5G). New sgRNAs targeting *NK4, Chordin,* and *Msx* were designed using CRISPOR [70,71], and validated by Amplicon E-Z sequencing service by Genewiz/Azenta (South Plainfield, NJ) as previously described [66,72]. A previously published *Msx*-targeting sgRNA [43] was also newly validated by Amplicon E-Z. The validation protocol can be followed at dx.doi.org/10.17504/protocols.io.14egnr97yl5d/v1 [66]. Cas9 was frequently fused to Geminin^N-terminus^, which increases mutagenesis efficacy [73,74]. All sgRNA, Cas9, primer, and relevant plasmid DNA sequences and amounts are included in the Supplemental Sequences file (S1 Text).

## Supporting information

S1 Video 3D projection of confocal image Z-stack through a larva co-electroporated with *NK4 int1.1 + bpFOG > GFP* (green) and *Foxg>H2B::mCherry* (magenta). Ventral medial papilla initially on the left, dorsal to the upper right.
(MP4)

S2 Video A Z-stack series from confocal imaging of an *NK4* CRISPR larva (using *Foxc>Cas9::Geminin*^*N-terminus*^), co-electroporated with *Seldom>Unc-76::GFP* (green cytoplasm) and *Foxc>H2B::mCherry* (red nuclei). Apical cilia are labeled by immunostaining for acetylated tubulin (AlexaFluor-488, also green), showing that PNs surrounding the single large papilla are still ciliated. Larva is counterstained with DAPI (blue). Anterior to upper left, dorsal on top.
(AVI)

S3 Video 3D projection of same larva as in S2 Video. (AVI)

S4 Video 3D projection of a confocal image stack through a negative control larva electroporated with Seldom>Unc-76::GFP (green cytoplasm) and Foxc>H2B::mCherry (red nuclei). Apical cilia are labeled by immunostaining for acetylated tubulin (AlexaFluor-488, also green). Larva is counterstained with DAPI (blue). Anterior to bottom right, ventral on left.
(AVI)

S1 File Supplemental figures S1–S11. (ZIP)

S1 Data Raw quantitative data and statistical test tables. (XLSX)

S1 Text Supplemental Sequences file. All relevant DNA and protein sequences used in this study, plus electroporation recipes.
(DOCX)
