# Supplementary material for: An NKX2-3/5/6 homolog is required downstream of BMP signaling to pattern the sensory-adhesive organ of a tunicate larva
Source: PLoS Genet. 2026 Oct 12;22(10):e1012334. doi: 10.1371/journal.pgen.1012334 (PMC13671656; doi:10.1371/journal.pgen.1012334)
Supplement: Supplementary file 3 [file pgen.1012334.s009.docx]

We thank the reviewers for their criticisms and suggestions. Please see below point-by-point replies to their comments.

**Reviewer #1:**

***“The bulk of the data are fluorescent images, and it is not clear whether the images come from epifluorescence or confocal microscopy. It is often difficult to understand the spatial configuration. I would advise the authors to add 3D reconstruction, different views, or at least interpretation schemes. For example in Fig 5B,D, it seems there is a lot of out-of-focus data.”***

The images are mostly epifuorescence. We clarify in the methods that all images are epifluorescence unless otherwise noted. However, we do have a confocal image of the basic papilla configuration show in Supplemental Figure 1 for reference and we now add new 3D interpretation diagrams in figures 2D and 5I. A 3D projection is also available for *NK4* and *Foxg* in situs (HCR) in figure 5A.

***“Also from Fig 5A, the authors state NK4 appears to be expressed at lower levels in more dorsal and lateral regions, especially near the two dorsolateral “spots” of Foxg expression corresponding to the two dorsal papillae. The images shown are not really convincing.”***

For this we now include confocal images of double (two-color) HCR in situs for *Foxg* and *NK4,* along with quantification in Figure 5 and supplemental figure S1.

***“Also Fig 7D and S5, the expression of Chordin at the midline is not clear (by the way, Chordin was rather reported to have a lateral expression domain).”***

Indeed, *Chordin* expression starts at the late gastrula stage in the lateral borders of the neural plate. These cells then intercalate at the dorsal midline as the neural plate rolls up to form the neural tube. The cells at the dorsal midline in the larva that we show in Figure 7A are descended from those lateral border cells. Critically, this is not new data, as the figure is simply included to show where these cells are located relative to the papillae. In fact, Liu et al. (2023) have previously described the expression of *Chordin* relative to the papilla territory in detail. Briefly, throughout development, *Chordin* is expressed lateral/posterior/dorsal to the papillae, always closest to the two presumptive, dorsal/lateral papillae and further from more medial/anterior/ventral cells. We now clarify this in the text, placing more emphasis on those published works.

***“The data fit with a model whereby NK4 is a direct target of BMP signaling and mediates its activity. The authors could directly test the epistatic relationship by combining BMP activation and NK4 CRISPR.”***

We have tested this epistatic interaction in part doing the inverse perturbations- BMP inhibition (Noggin overexpression) + NK4 overexpression (Figure 4J,K) However, it was shown in Roure et al. 2023 that early BMP administration results in loss of *Foxc* expression and papilla formation, while later administration eliminates the ventral papilla only. Those results are consistent with other studies (for instance Imai et al. 2012), which points to a more broadly ventralizing effect of BMP signaling, which can promote a ventral epidermis fate over a papilla fate. We show here that not all of this ventralizing force of BMP acts through NK4, as *NK4* CRISPR did not abolish the BMP-dependent expression of *Msx* in the ventral part of the papilla territory (Figure 4F,G). Because of this, and given the variability of BMP overactivation phenotypes, we did not pursue the converse epistasis test (BMP activation + NK4 inactivation).

***“The BMP/NK4/Msx model is presented as a morphogen-based spatial control of gene expression, opposed to a MEK/ERK temporal control of gene expression. However, the temporal aspect has been overlooked in the study. Could the timed overexpression of NK4 phenocopy the different phenotypes observed following BMP activation at different developmental times? The authors could try using different drivers (ie earlier and later compared to Foxc) at least for overexpression, but also possibly for CRISPR.”***

We now attempt to address the earlier overexpression of NK4, using *FOG>NK4,* but this resulted in severely malformed larvae lacking discernible papillae (Supplemental Figure S4D). For later overexpression unfortunately we were limited by the lack of suitable promoters. The only option for later expression would be the *Foxg* promoter (*Foxg>NK4)*, but the interpretation could be hampered by likely auto-repressive effect of NK4 on the promoter.

***“Previous reports placed Sp6/7/8(Zf220) as a key regulator of Foxg expression (especially the transition from a U to 3 future papillae). Expression of this gene needs to be examined in NK4 overexpression and CRISPR.”***

We did not have a suitable *Sp6/7/8* reporter to examine this easily, but here we now report the result of our Sp6/7/8 perturbations on *Foxg* reporter expression (Figure 6I-K). Unexpectedly, Sp6/7/8 overexpression did not abolish or reduce *Foxg* reporter expression. Also, *Sp6/7/8* CRISPR did not phenocopy the BMP inhibition or *NK4* CRISPR, resulting in a rather flat, though *Foxg+,* papilla territory instead of a *Cyrano*-type single protrusion. More careful investigation of Sp6/7/8 function will be required to fully understand its role in patterning the papilla, which we plan to do now as part of future studies. We speculate that Sp6/7/8 may regulate other factors that indirectly affect papilla patterning and maintenance of *Foxg* expression in different ways, but that the major factor initially downregulating *Foxg* downstream of BMP, restricting it to three dots, is indeed NK4 and not Sp6/7/8.

***- Chordin CRISPR. In the hypothesis Chordin regulates the shape of the BMP gradient, one could anticipate a dorsal shift in Msx expression. It does not seem to be the case following Chordin inactivation. Could the authors discuss this point? Also it would be important to better describe the differentiation phenotype of the papillae. Are dorsal papillae missing or only Foxg reporter expression?***

Actually, we did notice anecdotally a shift in *Msx* expression in the *Chordin* CRISPR but did not previously measure/report it. Here we now report our analysis of this expansion- *Msx* reporter was significantly expanded in the *Chordin* CRISPR (Supplemental Figure S8B). As for the dorsal papillae, indeed the dorsal papillae were missing- we scored for dorsal papilla presence/absence, which we could ascertain by both brightfield and *Foxg>GFP* visualization. We describe this better in the text and figure legend now.

***“An interesting way of validating their model would be to mutate Msx using CRISPR/Cas9. The predicted phenotype is an absence of the ventral papilla. Also, does Msx overexpression lead to any phenotype?”***

Indeed, Msx alone promotes and expands *Foxg* expression throughout the papilla territory, as we show now in Figure 7H. However, the morphology of the single papilla is not protrusive, though not as flat as NK4 overexpression. As for the CRISPR, we initially did not have any potent sgRNAs targeting *Msx.* A previously published sgRNA (Coppola et al. 2020) when validated gave a low mutagenesis efficacy rate. In response to the reviewer’s comments, we have designed and validated two new sgRNAs targeting *Msx.* However, neither the two new ones used in combination or the old sgRNA resulted in loss of *Foxg* and ventral papilla formation. We have thus revised our model to say that, although Msx can suppress NK4 function and promote *Foxg* expression, it is not necessary for ventral papilla formation. This suggests additional regulatory mechanisms are involved in ventral papilla formation, in addition to Msx. It is also possible that Msx helps maintain *Foxg* expression in the ventral papilla later. We did notice a lack of *NK4* HCR signal in the ventral papilla late, which is now shown as a new figure in Supplemental Figure S1C. However, we now also show that Msx overexpression does not abolish *NK4>GFP* reporter (Supplemental Figure S9B,C). Therefore, further follow up studies would be needed to more carefully investigate the function of Msx and how the ventral papilla forms.

***“The number of independent experiments for each result is not indicated.”***

We indicate that most scoring experiments have been done in duplicate now, and we average the frequencies across both duplicates (error bars indicate upper and lower range of frequencies). We also ran Fisher’s exact tests on scoring data per duplicate, now all reported in Supplemental data S1 file.

***“Figure 7A,B: show control and Foxc>Msx alone.”***

We now show this as Figure 7H.

**“*NK4 overexpression (Foxc>NK4) leads to a flattened epithelium instead of papillae with a lack of Foxg expression. But Islet is still expressed. It would be important to know whether markers for other papillae cell types are also downregulated.”***

We tested this suggestion, and found that NK4 overexpression completely abolishes all the cell type-specific differentiation markers tested: *Seldom (KH.C4.78), C11.360, CryBG,* and *KH.L141.36.* We show this now as part of Supplemental Figure S4. However, loss of these markers may have more to do with blocking initial activation of *Foxg* in the first place, which may block papilla specification altogether, and may not reflect any endogenous role for NK4 in regulating these marker genes. However, this makes *Islet* the only marker downstream of Foxg that we still see expressed upon NK4 overexpression. What this means we do not know. NK4 is also expressed in the cardiopharyngeal mesoderm, where *Islet* is also expressed. It is possible that there are additional regulatory connections between NK4 and Islet that are not normally operating in the papillae but that we have triggered inadvertently with the *Foxc>NK4* plasmid. This may be a future direction for our research.

**Reviewer #2:**

***“(1) The spatial pattern of BMP signaling is central to the proposed mechanism. The authors should provide direct evidence demonstrating the spatial distribution of active BMP signaling relative to NK4-positive cells. In particular, data supporting the proposed complementary or dual distribution would significantly strengthen the model.”***

To clarify, the spatial distribution of BMP signaling has already been meticulously documented in Waki et al. 2015 Roure et al. 2023 that such data would be duplicative in our opinion. The regulatory connection between BMP and NK4 has also been demonstrated though not emphasized in the context of the papillae. We now better explain these background data and published references with changes to the text.

***“(2) In Figure 5A, the authors state that “At the initial tailbud stage, NK4 appeared to be most strongly expressed in Foxg-negative cells surrounding the future papillae.” However, based on the current images, NK4 and Foxg signals appear to overlap, making this conclusion difficult to support. Higher-resolution imaging or an independent approach, such as HCR, would be necessary to clearly demonstrate the spatial relationship between NK4 and Foxg expression.”***

***“(3) In Figure 7, the authors show that Msx overexpression can override NK4 activity and rescue Foxg expression and papilla development. However, it remains unclear whether endogenous Msx on the ventral side normally functions to antagonize NK4 activity. To strengthen this conclusion, I recommend performing loss-of-function experiments, such as CRISPR/Cas9-mediated disruption of endogenous Msx, to assess its requirement in this context.”***

As explained in the reply to reviewer 1, we have now done this using two new validated sgRNAs to complement a published but less efficacious sgRNA, and the result was that *Msx* does not appear to be required for *Foxg* expression ventrally nor the formation of the ventral papilla. However, we show that overexpression of Msx alone can also promote *Foxg* expression, expanding it in the papilla region though not quite promoting a large protrusion (instead a somewhat enlarged but blunt papilla, see additions to Figure 6 now). We have revised our proposed model in light of these data, proposing that Msx might act in parallel to other unknown factors, and that further work will be needed to elucidate the endogenous role of Msx.

***“(4) Statistical analysis is missing from all bar graphs. Although some trends are visually apparent, appropriate statistical tests should be performed for all quantitative data. P-values or significance indicators should be included in the figures, along with a description of the statistical methods in the Methods section.”***

We have now used the Fisher’s Exact Test for all bar graphs representing frequency proportions, those p-values can be found in the Supplemental Data file with all the numerical data. Tests were run on duplicates separately, not on the averages between duplicates. However, error bars are now shown on the plots representing upper and lower range limits of the frequencies plotted between duplicates.

***(5) Please provide the results of the corresponding control experiments for Figure 4E.***

***“(6) For Figure 7E, please provide evidence addressing whether Chordin overexpression affects NK4 expression levels.“***

We now show results of quantification of *NK4>GFP* in Chordin and Noggin overexpression conditions in Supplemental Figure S6. We have separate experiments normalizing by either FoxC>H2B::mCherry or Islet>mCherry (similar to Figure 4B-E). It is clear now that Chordin represses *NK4* reporter expression in a manner comparable to Noggin.

***“(7) Figures 5F and 5G demonstrate regulation of Msx expression by BMP signaling. However, the functional relevance of Msx is not fully established until the later results section. To improve narrative coherence, I suggest moving the data from Figures 5F and 5G to Figure 7.”***

To clarify, *Msx* as a BMP-responsive ventral papilla marker was previously established by Roure et al. 2023. We apologize for the incoherent narrative based on lack of sufficient background exposition. We now alter the text to more carefully explain the logic of looking at *Msx* in Figure 5 before later testing the functional significance of Msx.

***(8) In Figure 7D, an asterisk marks Chordin expression in migratory mesenchymal cells, but the biological significance of this observation is not clearly explained. Please clarify whether this is intended to demonstrate faithful recapitulation of endogenous Chordin expression by the transgene, or whether it has additional functional implications.***

By *in situ* hybridization as seen on GHOST or ANISEED databases, it appears *Chordin* is expressed in mesenchyme cells earlier (pre-migration). So, this reporter activity could be faithful recapitulation. However, we do not ascribe any functional implication to this expression. As expression would be much earlier, before the cells reach the anterior papilla region, we doubt these cells are releasing Chordin in the vicinity of the papillae, but rather in the posterior where they are located before the larval stages.

***“(9) In Figure 5C, the two categories are represented using very similar color schemes, making it difficult to distinguish between them. Please revise the color scheme to improve clarity and visual accessibility.”***

Fixed it.

***Reviewer #3:***

***“As a suggestion to further strengthen the Discussion, the authors may wish to consider explicitly engaging with the evolutionary implications of the present findings in relation to the anterior neural ridge/border (ANR/ANB) and its vertebrate placode homologs. Such a discussion would enhance the broader significance of the work.”***

We wished to defer to Roure et al. and Liu et al. which discuss in detail the evolutionary implications of BMP at the ANB, and we continue to do so- but where we think we can add to the discussion is specifically the BMP-NK4 connection. Previously we were unaware of any such connections in vertebrates, but following the reviewer’s suggestion, we dug deeper into the literature and found specific roles for Nkx2.3 in ectodermal organ development, like salivary glands and teeth. The evolutionary implications are intriguing, and we discuss these now in the last paragraph of the discussion. BMP is also involved in salivary gland and tooth development, but there is yet, to our knowledge, any firm link connecting BMP to upregulation of Nkx2.3 in these contexts. This may be interesting for those studying vertebrate orofacial ectoderm to pursue in the future. Here, we added new data to show that all three human paralogs (NKX2-3, NKX2-5, and NKX2-6) repress *Foxg* and papilla formation when overexpressed in *Ciona*.

***“On a related note, it would be of interest — even if purely speculative at this stage — to consider species such as Styela clava (Styelidae), in which larvae reportedly possess a single papilla rather than the tripartite arrangement typical of most ascidians. In light of the current model, one might ask whether altered NK4 expression domains, or changes in the relative activities of Chordin and Msx, could account for such inter-species variation in papilla number. Even a brief consideration of this question would lend additional evolutionary depth to an already strong manuscript.”***

We thank the reviewer for their words of support and their interesting suggestion. We have added such speculation to the discussion now as well. In our minds, there are two possibilities- one is that NK4 function/expression is altered in those such that they have lost tripartite patterning (tripartite organization appears to be the ancestral condition, at least in extant ascidians). Alternatively, there is still a cryptic tripartite patterning of gene expression and/or cell types, even if there is obvious tripartite morphology. It will indeed be interesting to consider these possibilities.
