# Supplementary material for: An NKX2-3/5/6 homolog is required downstream of BMP signaling to pattern the sensory-adhesive organ of a tunicate larva
Source: PLoS Genet. 2026 Oct 12;22(10):e1012334. doi: 10.1371/journal.pgen.1012334 (PMC13671656; doi:10.1371/journal.pgen.1012334)
Supplement: Supplementary file 5 [file pgen.1012334.s008.docx]

In this manuscript, Johnson et.al build upon prior work to elucidate the mechanisms governing the spatial patterning and numerical specification of the sensory adhesive organs (papillae) in ascidian larvae. While previous studies have broadly characterized cell fate specification within the anterior neural plate, the precise regulatory logic dictating the formation of these three distinct papillary units has remained incompletely understood. Using a rigorous combination of tissue-specific overexpression and CRISPR/Cas9-mediated knockdown, the authors demonstrate that the transcription factor *NK4* acts as a repressor of *Foxg*, a master regulator of papilla fate. Furthermore, they map a regulatory network wherein BMP signaling drives *NK4* expression, which is subsequently spatially restricted by the *Msx* transcription factor, ultimately confining *Foxg* expression to three discrete domains. I believe this study is significant progress in our understanding of papillary patterning. Furthermore, since the anterior border of the neural plate is evolutionarily linked to vertebrate placodes, investigating its embryonic origin, patterning mechanisms, and molecular signatures provides crucial insights into evolutionary biology. Therefore, this work has the potential to impact a broad range of research fields.

Overall, the experimental data are compelling and largely substantiate the authors’ proposed regulatory model. However, to fully support their conclusions, the manuscript requires additional functional evidence and spatial resolution.

**(1)** The spatial pattern of BMP signaling is central to the proposed mechanism. The authors should provide direct evidence demonstrating the spatial distribution of active BMP signaling relative to NK4-positive cells. In particular, data supporting the proposed complementary or dual distribution would significantly strengthen the model.

**(2)** In Figure 5A, the authors state that “At the initial tailbud stage, NK4 appeared to be most strongly expressed in Foxg-negative cells surrounding the future papillae.” However, based on the current images, NK4 and Foxg signals appear to overlap, making this conclusion difficult to support. Higher-resolution imaging or an independent approach, such as HCR, would be necessary to clearly demonstrate the spatial relationship between NK4 and Foxg expression.

**(3)** In Figure 7, the authors show that Msx overexpression can override NK4 activity and rescue Foxg expression and papilla development. However, it remains unclear whether endogenous Msx on the ventral side normally functions to antagonize NK4 activity. To strengthen this conclusion, I recommend performing loss-of-function experiments, such as CRISPR/Cas9-mediated disruption of endogenous Msx, to assess its requirement in this context.

**(4)** Statistical analysis is missing from all bar graphs. Although some trends are visually apparent, appropriate statistical tests should be performed for all quantitative data. P-values or significance indicators should be included in the figures, along with a description of the statistical methods in the Methods section.

**(5)** Please provide the results of the corresponding control experiments for Figure 4E.

**(6)** For Figure 7E, please provide evidence addressing whether Chordin overexpression affects NK4 expression levels.

**(7)** Figures 5F and 5G demonstrate regulation of Msx expression by BMP signaling. However, the functional relevance of Msx is not fully established until the later results section. To improve narrative coherence, I suggest moving the data from Figures 5F and 5G to Figure 7.

**(8)** In Figure 7D, an asterisk marks Chordin expression in migratory mesenchymal cells, but the biological significance of this observation is not clearly explained. Please clarify whether this is intended to demonstrate faithful recapitulation of endogenous Chordin expression by the transgene, or whether it has additional functional implications.

**(9)** In Figure 5C, the two categories are represented using very similar color schemes, making it difficult to distinguish between them. Please revise the color scheme to improve clarity and visual accessibility.
