# Supplementary material for: An NKX2-3/5/6 homolog is required downstream of BMP signaling to pattern the sensory-adhesive organ of a tunicate larva
Source: PLoS Genet. 2026 Oct 12;22(10):e1012334. doi: 10.1371/journal.pgen.1012334 (PMC13671656; doi:10.1371/journal.pgen.1012334)
Supplement: Peer review history

# Peer review: Decision Letter 0

**DOI:** 10.1371/journal.pgen.1012334.r001

**Authors:** Fengwei Yu, Nandan Nerurkar

15 Apr 2026

PGENETICS-D-25-01397

An NKX2-5 homolog is required downstream of BMP signaling to pattern the sensory-adhesive organ of a tunicate larva

PLOS Genetics

Dear Dr. Stolfi,

Thank you for submitting your manuscript to PLOS Genetics. After careful consideration, we feel that it has merit but does not fully meet PLOS Genetics's publication criteria as it currently stands. Therefore, we invite you to submit a revised version of the manuscript that addresses the points raised during the review process.

Please submit your revised manuscript by Jun 14 2026 11:59PM. If you will need more time than this to complete your revisions, please reply to this message or contact the journal office at plosgenetics@plos.org. When you're ready to submit your revision, log on to https://www.editorialmanager.com/pgenetics/ and select the 'Submissions Needing Revision' folder to locate your manuscript file.

Please include the following items when submitting your revised manuscript:

* A letter that responds to each point raised by the editor and reviewer(s). You should upload this letter as a separate file labeled 'Response to Reviewers'. This file does not need to include responses to any formatting updates and technical items listed in the 'Journal Requirements' section below.

* A marked-up copy of your manuscript that highlights changes made to the original version. You should upload this as a separate file labeled 'Revised Manuscript with Track Changes'.

* An unmarked version of your revised paper without tracked changes. You should upload this as a separate file labeled 'Manuscript'.

If you would like to make changes to your financial disclosure, competing interests statement, or data availability statement, please make these updates within the submission form at the time of resubmission. Guidelines for resubmitting your figure files are available below the reviewer comments at the end of this letter.

We look forward to receiving your revised manuscript.

Kind regards,

Nandan Nerurkar

Academic Editor

PLOS Genetics

Fengwei Yu

Section Editor

PLOS Genetics

Aimée Dudley

Editor-in-Chief

PLOS Genetics

Anne Goriely

Editor-in-Chief

PLOS Genetics

**Journal Requirements:**

1) We ask that a manuscript source file is provided at Revision. Please upload your manuscript file as a .doc, .docx, .rtf or .tex. If you are providing a .tex file, please upload it under the item type u2018LaTeX Source Fileu2019 and leave your .pdf version as the item type u2018Manuscriptu2019.

2) Please provide an Author Summary. This should appear in your manuscript between the Abstract (if applicable) and the Introduction, and should be 150-200 words long. The aim should be to make your findings accessible to a wide audience that includes both scientists and non-scientists. Sample summaries can be found on our website under Submission Guidelines:

https://journals.plos.org/plosgenetics/s/submission-guidelines#loc-parts-of-a-submission

3) Please upload all main figures as separate Figure files in .tif or .eps format. For more information about how to convert and format your figure files please see our guidelines:

https://journals.plos.org/plosgenetics/s/figures

4) We notice that your supplementary Figures are included in the manuscript file. Please remove them and upload them with the file type 'Supporting Information'. Please ensure that each Supporting Information file has a legend listed in the manuscript after the references list.

5) Please amend your detailed Financial Disclosure statement. This is published with the article. It must therefore be completed in full sentences and contain the exact wording you wish to be published.

State what role the funders took in the study. If the funders had no role in your study, please state: "The funders had no role in study design, data collection and analysis, decision to publish, or preparation of the manuscript.".

If you did not receive any funding for this study, please simply state: u201cThe authors received no specific funding for this work.u201d

**Reviewers' comments:**

Reviewer's Responses to Questions

**Comments to the Authors:**

**Please note here if the review is uploaded as an attachment.**

Reviewer #1: Johnson et al. study the function of the gene NK4, the ortholog of the Nkx2-5 gene, in the patterning of the papillae, a sensory and adhesive organ located at the anterior edge of ascidian larvae. Using a combination of gain- and loss-of-function together with gene expression analyses, they show that NK4 acts downstream of BMP signaling to regulate Foxg expression and subsequent formation of three separate papillae. This study is solid and nicely performed, and allows to propose of gradient model for papillae formation. Yet, some elements are needed to support the claims made by the authors:

- The bulk of the data are fluorescent images, and it is not clear whether the images come from epifluorescence or confocal microscopy. It is often difficult to understand the spatial configuration. I would advise the authors to add 3D reconstruction, different views, or at least interpretation schemes. For example in Fig 5B,D, it seems there is a lot of out-of-focus data. Also from Fig 5A, the authors state NK4 appears to be expressed at lower levels in more dorsal and lateral regions, especially near the two dorsolateral “spots” of Foxg expression corresponding to the two dorsal papillae. The images shown are not really convincing. Also Fig 7D and S5, the expression of Chordin at the midline is not clear (by the way, Chordin was rather reported to have a lateral expression domain).

- The data fit with a model whereby NK4 is a direct target of BMP signaling and mediates its activity. The authors could directly test the epistatic relationship by combining BMP activation and NK4 CRISPR.

- The BMP/NK4/Msx model is presented as a morphogen-based spatial control of gene expression, opposed to a MEK/ERK temporal control of gene expression. However, the temporal aspect has been overlooked in the study. Could the timed overexpression of NK4 phenocopy the different phenotypes observed following BMP activation at different developmental times? The authors could try using different drivers (ie earlier and later compared to Foxc) at least for overexpression, but also possibly for CRISPR.

- Previous reports placed Sp6/7/8(Zf220) as a key regulator of Foxg expression (especially the transition from a U to 3 future papillae). Expression of this gene needs to be examined in NK4 overexpression and CRISPR.

- Chordin CRISPR. In the hypothesis Chordin regulates the shape of the BMP gradient, one could anticipate a dorsal shift in Msx expression. It does not seem to be the case following Chordin inactivation. Could the authors discuss this point? Also it would be important to better describe the differentiation phenotype of the papillae. Are dorsal papillae missing or only Foxg reporter expression?

- An interesting way of validating their model would be to mutate Msx using CRISPR/Cas9. The predicted phenotype is an absence of the ventral papilla. Also, does Msx overexpression lead to any phenotype?

Minor points:

- The number of independent experiments for each result is not indicated.

- Figure 7A,B: show control and Foxc>Msx alone.

- NK4 overexpression (Foxc>NK4) leads to a flattened epithelium instead of papillae with a lack of Foxg expression. But Islet is still expressed. It would be important to know whether markers for other papillae cell types are also downregulated.

Reviewer #2: the review is uploaded as an attachment

Reviewer #3: This manuscript addresses the patterning mechanism of the sensory-adhesive papillae — three anteriorly positioned organs in the tunicate larva that serve as the primary mechanosensory trigger for metamorphosis initiation. The authors demonstrate, through systematic perturbation of upstream and downstream components of the BMP signaling pathway, that the NKX2-3/5/6 ortholog NK4 acts as a transcriptional repressor downstream of BMP to pattern the papilla territory into its characteristic tripartite arrangement. The evidence presented is logically organized and collectively provides compelling support for the proposed regulatory model: BMP activates NK4 expression throughout much of the papilla territory, while dorsal Chordin restricts NK4 dorsally to permit formation of the two dorsal papillae, and ventral Msx antagonizes NK4 function to allow emergence of the single ventral papilla.

The experimental design is rigorous and the data presented at each step substantiate the authors' claims within each section. The Discussion effectively situates these findings within the classical BMP-Chordin morphogen gradient framework and advances an evolutionarily meaningful and intellectually compelling model for tripartite papilla patterning.

As a suggestion to further strengthen the Discussion, the authors may wish to consider explicitly engaging with the evolutionary implications of the present findings in relation to the anterior neural ridge/border (ANR/ANB) and its vertebrate placode homologs. Such a discussion would enhance the broader significance of the work. On a related note, it would be of interest — even if purely speculative at this stage — to consider species such as Styela clava (Styelidae), in which larvae reportedly possess a single papilla rather than the tripartite arrangement typical of most ascidians. In light of the current model, one might ask whether altered NK4 expression domains, or changes in the relative activities of Chordin and Msx, could account for such inter-species variation in papilla number. Even a brief consideration of this question would lend additional evolutionary depth to an already strong manuscript.

**********

**Have all data underlying the figures and results presented in the manuscript been provided?**

Large-scale datasets should be made available via a public repository as described in the *PLOS Genetics*  data availability policy, and numerical data that underlies graphs or summary statistics should be provided in spreadsheet form as supporting information.

Reviewer #1: Yes

Reviewer #2: Yes

Reviewer #3: Yes

**********

PLOS authors have the option to publish the peer review history of their article (what does this mean?). If published, this will include your full peer review and any attached files.

If you choose “no”, your identity will remain anonymous but your review may still be made public.

**Do you want your identity to be public for this peer review?**  For information about this choice, including consent withdrawal, please see our Privacy Policy.

Reviewer #1: No

Reviewer #2: **Yes:**  Chen Cao

Reviewer #3: **Yes:**  Kohji Hotta

[NOTE: If reviewer comments were submitted as an attachment file, they will be attached to this email and accessible via the submission site. Please log into your account, locate the manuscript record, and check for the action link "View Attachments". If this link does not appear, there are no attachment files.]

**Figure resubmission:**

**Reproducibility:**

To enhance the reproducibility of your results, we recommend that authors of applicable studies deposit laboratory protocols in protocols.io, where a protocol can be assigned its own identifier (DOI) such that it can be cited independently in the future. Additionally, PLOS ONE offers an option to publish peer-reviewed clinical study protocols. Read more information on sharing protocols at https://plos.org/protocols?utm_medium=editorial-email&utm_source=authorletters&utm_campaign=protocols

Attachment Submitted filename: PLos_review_cc.docx

# Author comment: Author response to Decision Letter 1

**DOI:** 10.1371/journal.pgen.1012334.r002

11 Aug 2026

Attachment Submitted filename: Replies-to-Reviewers-NK4-080626.docx

# Peer review: Decision Letter 1

**DOI:** 10.1371/journal.pgen.1012334.r003

**Authors:** Fengwei Yu, Nandan Nerurkar

27 Sep 2026

Dear Dr Stolfi,

We are pleased to inform you that your manuscript entitled "An NKX2-3/5/6 homolog is required downstream of BMP signaling to pattern the sensory-adhesive organ of a tunicate larva" has been editorially accepted for publication in PLOS Genetics. Congratulations!

Before your submission can be formally accepted and sent to production you will need to complete our formatting changes, which you will receive in a follow up email. Please be aware that it may take several days for you to receive this email; during this time no action is required by you. Please note: the accept date on your published article will reflect the date of this provisional acceptance, but your manuscript will not be scheduled for publication until the required changes have been made.

Once your paper is formally accepted, an uncorrected proof of your manuscript will be published online ahead of the final version, unless you’ve already opted out via the online submission form. If, for any reason, you do not want an earlier version of your manuscript published online or are unsure if you have already indicated as such, please let the journal staff know immediately at plosgenetics@plos.org.

In the meantime, please log into Editorial Manager at https://www.editorialmanager.com/pgenetics/, click the "Update My Information" link at the top of the page, and update your user information to ensure an efficient production and billing process. Note that PLOS requires an ORCID iD for all corresponding authors. Therefore, please ensure that you have an ORCID iD and that it is validated in Editorial Manager. To do this, go to ‘Update my Information’ (in the upper left-hand corner of the main menu), and click on the Fetch/Validate link next to the ORCID field.  This will take you to the ORCID site and allow you to create a new iD or authenticate a pre-existing iD in Editorial Manager.

If you have a press-related query, or would like to know about making your underlying data available (as you will be aware, this is required for publication), please see the end of this email. If your institution or institutions have a press office, please notify them about your upcoming article at this point, to enable them to help maximise its impact. Inform journal staff as soon as possible if you are preparing a press release for your article and need a publication date.

Thank you again for supporting open-access publishing; we are looking forward to publishing your work in PLOS Genetics!

Yours sincerely,

Nandan Nerurkar

Academic Editor

PLOS Genetics

Fengwei Yu

Section Editor

PLOS Genetics

Aimée Dudley

Editor-in-Chief

PLOS Genetics

Anne Goriely

Editor-in-Chief

PLOS Genetics

www.plosgenetics.org

BlueSky: @plos.bsky.social

----------------------------------------------------

Comments from the reviewers (if applicable):

Reviewer's Responses to Questions

**Comments to the Authors:**

**Please note here if the review is uploaded as an attachment.**

Reviewer #1: The study presented in the revision is greatly improved.

Only two minor points:

- clarify Foxg expression in Sp6/7/8/9 CRISPR: expressed at same levels? same pattern (3 spots or continous U-shaped stripe)?

- Fig S5B: swap gray bar between control and NKX2-5 overexpression.

Reviewer #2: The authors have addressed all of my concerns, and I recommend the manuscript for publication.

Reviewer #3: The authors addressed my previous requests. The evolutionary discussion and the newly added experiment examining the effects of the three human NKX2 paralogs substantially strengthen the manuscript.The newly added evolutionary discussion will enhance the more evolutionary implications. I have no further substantive commnts.

**********

**Have all data underlying the figures and results presented in the manuscript been provided?**

Large-scale datasets should be made available via a public repository as described in the *PLOS Genetics*  data availability policy, and numerical data that underlies graphs or summary statistics should be provided in spreadsheet form as supporting information.

Reviewer #1: Yes

Reviewer #2: Yes

Reviewer #3: Yes

**********

PLOS authors have the option to publish the peer review history of their article (what does this mean?). If published, this will include your full peer review and any attached files.

If you choose “no”, your identity will remain anonymous but your review may still be made public.

**Do you want your identity to be public for this peer review?**  For information about this choice, including consent withdrawal, please see our Privacy Policy.

Reviewer #1: No

Reviewer #2: **Yes:**  Chen Cao

Reviewer #3: **Yes:**  Kohji Hotta

----------------------------------------------------

**Data Deposition**

If you have submitted a Research Article or Front Matter that has associated data that are not suitable for deposition in a subject-specific public repository (such as GenBank or ArrayExpress), one way to make that data available is to deposit it in the Dryad Digital Repository. As you may recall, we ask all authors to agree to make data available; this is one way to achieve that. A full list of recommended repositories can be found on our website.

The following link will take you to the Dryad record for your article, so you won't have to re‐enter its bibliographic information, and can upload your files directly:

http://datadryad.org/submit?journalID=pgenetics&manu=PGENETICS-D-25-01397R1

More information about depositing data in Dryad is available at http://www.datadryad.org/depositing. If you experience any difficulties in submitting your data, please contact help@datadryad.org for support.

Additionally, please be aware that our data availability policy requires that all numerical data underlying display items are included with the submission, and you will need to provide this before we can formally accept your manuscript, if not already present.

----------------------------------------------------

**Press Queries**

If you or your institution will be preparing press materials for this manuscript, or if you need to know your paper's publication date for media purposes, please inform the journal staff as soon as possible so that your submission can be scheduled accordingly. Your manuscript will remain under a strict press embargo until the publication date and time. This means an early version of your manuscript will not be published ahead of your final version. PLOS Genetics may also choose to issue a press release for your article. If there's anything the journal should know or you'd like more information, please get in touch via plosgenetics@plos.org.

# Editor report: Acceptance letter

**DOI:** 10.1371/journal.pgen.1012334.r004

**Authors:** Fengwei Yu, Nandan Nerurkar

PGENETICS-D-25-01397R1

An NKX2-3/5/6 homolog is required downstream of BMP signaling to pattern the sensory-adhesive organ of a tunicate larva

Dear Dr Stolfi,

We are pleased to inform you that your manuscript entitled "An NKX2-3/5/6 homolog is required downstream of BMP signaling to pattern the sensory-adhesive organ of a tunicate larva" has been formally accepted for publication in PLOS Genetics! Your manuscript is now with our production department and you will be notified of the publication date in due course.

The corresponding author will soon be receiving a typeset proof for review, to ensure errors have not been introduced during production. Please review the PDF proof of your manuscript carefully, as this is the last chance to correct any errors. Please note that major changes, or those which affect the scientific understanding of the work, will likely cause delays to the publication date of your manuscript.

Soon after your final files are uploaded, unless you have opted out or your manuscript is a front-matter piece, the early version of your manuscript will be published online. The date of the early version will be your article's publication date. The final article will be published to the same URL, and all versions of the paper will be accessible to readers.

For Research Articles, you will receive an invoice from PLOS for your publication fee after your manuscript has reached the completed accept phase. If you receive an email requesting payment before acceptance or for any other service, this may be a phishing scheme. Learn how to identify phishing emails and protect your accounts at https://explore.plos.org/phishing.

Thank you again for supporting PLOS Genetics and open-access publishing. We are looking forward to publishing your work!

With kind regards,

Janani Seenivasan

PLOS Genetics

On behalf of:

The PLOS Genetics Team

Carlyle House, Carlyle Road, Cambridge CB4 3DN | United Kingdom

plosgenetics@plos.org | +44 (0) 1223-442823

plosgenetics.org | Twitter: @PLOSGenetics
